# Mechanobiological Dynamics‐Inspired Mechanomodulatory Biomaterials

**DOI:** 10.1002/advs.202416992

**Published:** 2025-12-08

**Authors:** Letao Yang, Pengfei Jiang, Joshua B Stein, Yannan Hou, Chaochen Zhou, Heemin Kang, Ki‐Bum Lee

**Affiliations:** ^1^ Shanghai Tongji Hospital Key Laboratory of Spine and Spinal Cord Injury Repair and Regeneration Ministry of Education Frontier Science Center for Stem Cell Research School of Life Sciences and Technology Tongji University Shanghai 200092 China; ^2^ Department of Chemistry and Chemical Biology Rutgers University Piscataway NJ 08854 USA; ^3^ Department of Materials Science and Engineering Korea University Seoul 02841 South Korea

**Keywords:** artificial intelligence, biomaterials, mechanotransduction, nanobiotechnology, nanomedicine, stem cells

## Abstract

Mechanical cues are fundamental regulators of stem cell fate and play critical roles in various biological processes, including embryogenesis, tissue repair, and regeneration. Successfully reconstructing the complex and dynamic mechanical microenvironments of human tissues necessitates innovative biomaterial designs that surpass conventional approaches. This review provides a comprehensive overview of recent advances in the field of biomaterial‐mediated mechanomodulation of stem cell fate, encompassing both mechanobiological dynamics and dynamic mechanomodulatory biomaterials. It is also discussed how specific material properties, such as stiffness, nanotopography, shear stress, and dynamic stimuli‐responsive behavior, can be used to precisely control stem cell processes, including proliferation, differentiation, migration, and apoptosis. Furthermore, the application of these strategies is examined in both conventional and advanced culture systems, such as organoids and organ‐on‐chip platforms, with a particular focus on tissue‐engineering applications in the neurological, musculoskeletal, and endocrine systems. It is further discussed how material innovations have enabled the development of cutting‐edge techniques for investigating mechanotransduction in stem cells, including force probes, non‐invasive biosensors, materiomics, and machine learning. By integrating knowledge from diverse fields, including medicine, materials science, engineering, biology, and biophysics, this review ultimately aims to inspire the design of smarter biomaterial systems that can accelerate the clinical translation of mechanotherapies and advance the field of regenerative medicine.

## Graphical abstract

Recent advances in biomaterial‐mediated mechanomodulation of stem cell fate, encompassing 2, 3, and 4D systems and their synergy with artificial intelligence is overviewed. By integrating knowledge from diverse fields, this review ultimately aims to inspire the design of smarter biomaterial systems that can accelerate the clinical translation of mechanotherapies and advance the field of regenerative medicine.

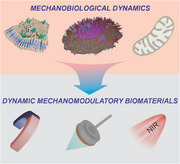

## 1 Introduction

In human tissues, cells constantly receive passive and active mechanical signals from surrounding cells, fluids, and extracellular matrix (ECM). For example, the mechanical cue mediated by the ECM defines the polarization of embryos and the formation of neural tubes by modulating stem cell differentiation and migration, respectively.^[^ ^1^, ^2^, ^3^ ^]^ Mechanical signals that dynamically remodel the proliferation, differentiation, migration, and apoptosis of stem cells, also profoundly influence tissue regeneration in human diseases.^[^ ^4^, ^5^ ^]^ Therefore, a fundamental goal in mechanobiology and tissue engineering is to elucidate the intricate mechanisms by which mechanical stimuli and forces affect the complex processes of stem cell differentiation and lineage commitment. Understanding these relationships would be crucial for developing more effective strategies in regenerative medicine and creating biomaterials that can precisely control stem cell fate. In addition, by unraveling the connections between mechanical cues and cellular responses, scientists can harness this knowledge to design advanced tissue engineering approaches and to manipulate stem cell behavior for therapeutic applications.

Mechanobiology mainly focuses on how biomacromolecules, organelles, cells, tissues, and organs respond to mechanical signals and how these responses affect development and physiology. Initially established a century ago, mechanobiology has witnessed great prosperity with the integration of the interdisciplinary fields of material science, medicine, and engineering into biology and biophysics.^[^ ^6^ ^]^ For instance, cellular reprogramming technologies, new mechanotransduction mechanisms, smart mechanomodulatory biomaterials, and novel mechanobiology tools for measuring cellular forces have all contributed exciting innovations to stem cell mechanobiology. Adapting the state‐of‐the‐art mechanotransduction mechanism and mechanobiology tools into biomaterial research would reshape the development of next‐generation mechanotherapy.

Early investigations into mechanical cues and mechanotransduction at 2D material‐stem cell interfaces proved instrumental in advancing our understanding of stem cell behavior and potential applications in regenerative medicine.^[^ ^7^ ^]^ Several seminal reviews have provided overviews of the important concepts of mechanotransduction pathways associated with stiffness,^[^ ^8^, ^9^, ^10^ ^]^ nanotopography,^[^ ^11^ ^]^ porosity,^[^ ^12^ ^]^ and adhesive ligand density.^[^ ^13^ ^]^ Following these initial works, significant progress was made in identifying new dimensions of mechanical cues. This included recognizing the role of viscoelasticity in cell‐matrix interactions,^[^ ^14^ ^]^ how cells generate traction forces in response to hydrogel degradation, and the impact of shear stress on cell behavior in dynamic organ‐on‐chip systems.^[^ ^15^ ^]^ Alongside these discoveries, a paradigm shift has occurred from 2D cell culture to 3D approaches that better recapitulate physiological environments. This includes in vitro systems such as stem cell‐encapsulated hydrogels and spheroids, and extends to the use of in vivo tissue models. This paradigm shift greatly improves the translation of mechanomodulatory materials for mechanotherapy. Hence, up‐to‐date reviews on the topic of 3D biomaterial‐mediated mechanomodulation of stem cells have also inspired novel designs of hydrogel^[^ ^16^ ^]^ and 3D scaffold biomaterials.^[^ ^17^, ^18^ ^]^ In parallel, the discovery of new mechanotransduction mechanisms fueled the field of mechanobiology, including the Hippo pathway and epigenetics, which also inspired the design of smart biomaterials.^[^ ^19^, ^20^, ^21^ ^]^ Other excellent review papers have also provided discussions and insights on these critical topics.

**Figure 1:**
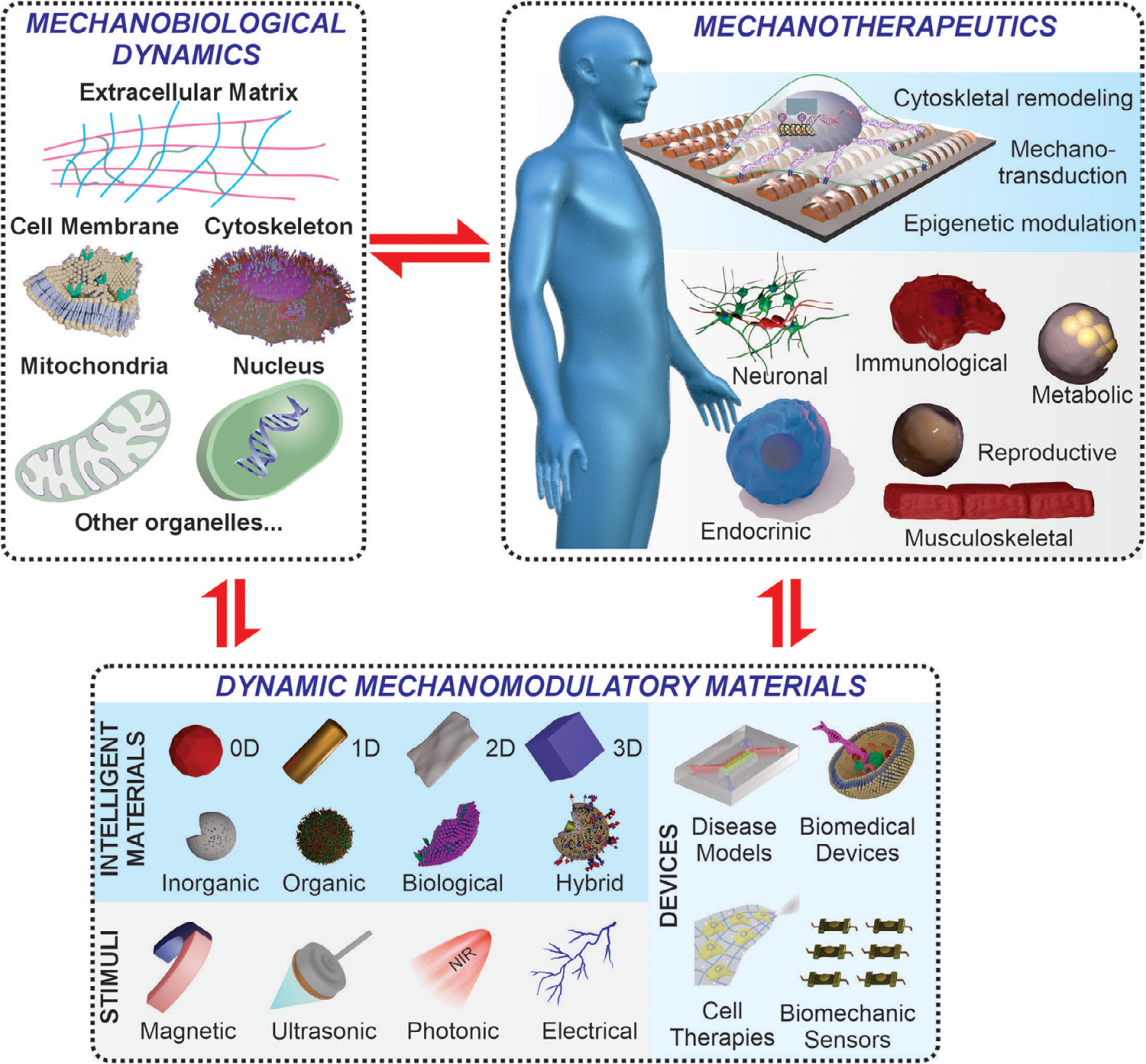
An overview of material intelligence and dynamics for advancing stem cell mechanobiology and therapy. The left panel summarizes the structure of the current review on the analysis of mechanobiological dynamics within different cell organelles and extracellular components. The bottom panel summarizes different categories of intelligent materials, stimuli‐responsive dynamic biomaterials, and devices that induce mechanotransduction in stem cells. The right panel summarizes the mechanotransduction processes in varying potential applications of mechanotherapeutics.

Despite these rapid advances in mechanobiology, smart biomaterial design, and bioengineering, there is a compelling need to cultivate a more forward‐thinking strategy concerning dynamic mechanomodulatory biomaterials. Incorporating the temporal changes of mechanotransduction into biomaterial design would not only be essential for understanding many of the developmental and disease processes, but also would lead to more effective and robust tissue engineering strategies. To this end, the current review will first summarize mechanobiological dynamics at the ECM and varying cell organelles, then introduce the design of stimuli‐responsive biomaterials for dynamic mechanomodulation of cell fates. Additionally, we will examine novel biomaterials and emerging techniques that enable deeper insights into mechanobiological dynamics. Finally, we will outline our perspectives on key future directions, including the integration with multi‐omics technologies and pathways toward clinical translation. Overall, by refining the state‐of‐the‐art knowledge from multidisciplinary fields of medicine, material science, engineering, biology, and biophysics, we aim to provide an up‐to‐date overview of the most important topics related to biomaterial‐based mechanomodulation and mechanotransduction to facilitate both the clinical translation and fundamental studies of stem cell mechanobiology (**Figure** **1**). Through these efforts, we also aim to inspire the design of smart biomaterial systems that can accelerate the discovery of novel mechanotherapies and regenerative medicine.

## 2 Overview of Mechanotransduction Pathways

**Figure 2:**
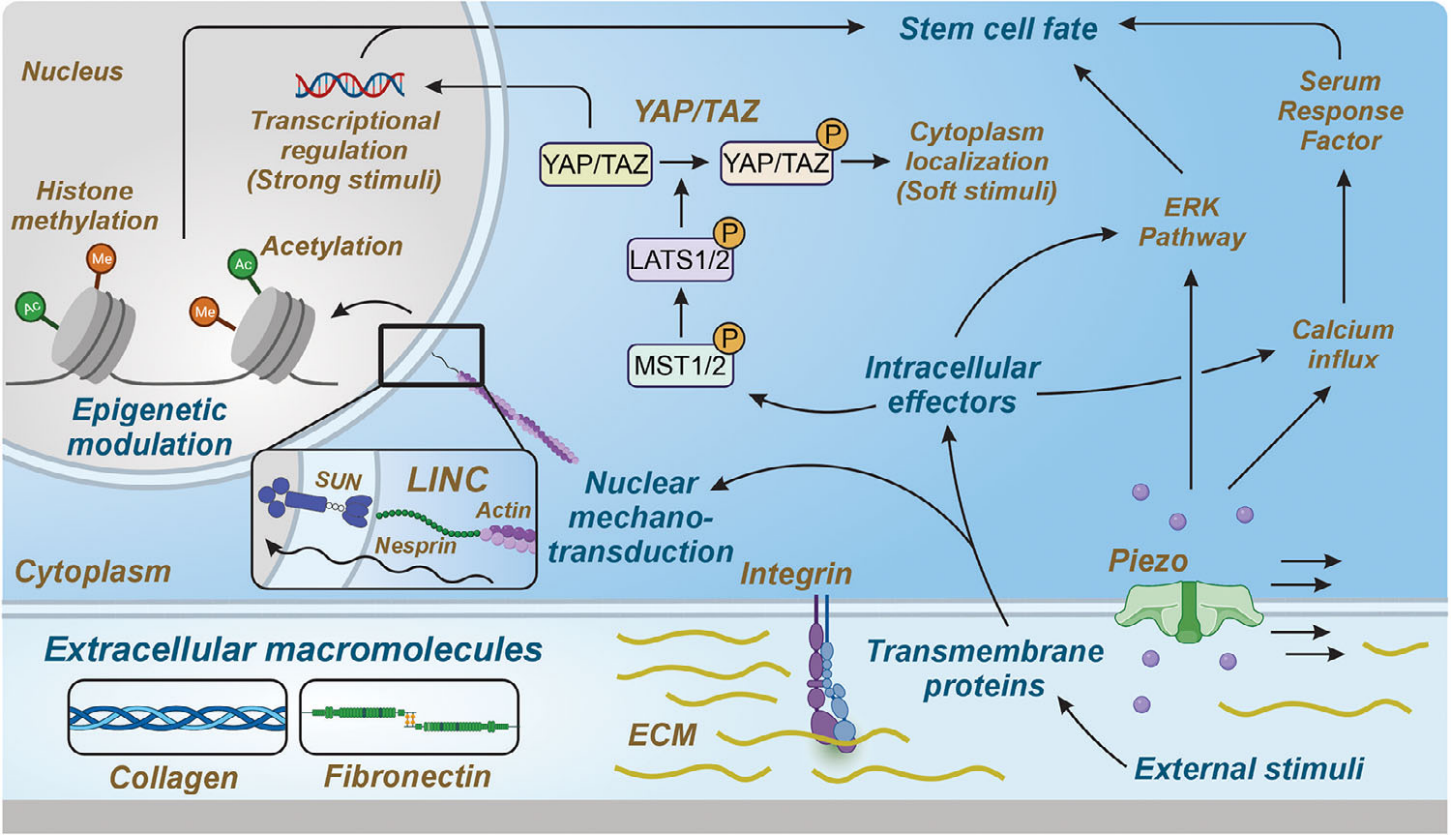
An overview of mechanotransduction pathways. The diagram illustrates how mechanical cues—such as extracellular matrix stiffness, shear stress, and tensile strain—are sensed by cells and transduced into biochemical signals. Key mechanosensors include integrins, stretch‐activated ion channels (e.g., Piezo1), and cell–cell adhesion complexes (e.g., cadherins). These inputs are transmitted via the cytoskeleton and focal adhesion complexes to intracellular signaling pathways, including RhoA/ROCK, FAK/Src, MAPK, and the YAP/TAZ‐mediated Hippo pathway. Signal integration at the nucleus leads to changes in gene expression that regulate cellular responses, such as migration, proliferation, differentiation, and apoptosis. This scheme emphasizes the coordinated interplay between mechanical stimuli and cellular signaling machinery.

Research in the past two decades has provided strong evidence for the role of mechanical signaling on cellular fate control, both at the developmental stages and after in mature adult tissues, including mitosis, proliferation, morphogenesis, migration, differentiation, fusion, and apoptosis (**Figure** **2**).^[^ ^22^ ^]^ There are generally two types of mechanical signaling, known as “outside‐in” and “inside‐out” signaling. “Outside‐in” signaling is often initiated by external forces that are either transmitted to the cell membranes or mediated by the properties of ECM, such as stiffness, nanotopography, shear stress, and viscoelasticity. In contrast, “inside‐out” signaling is controlled by altering the mechanical properties of the cell surfaces or cytoskeleton. Note that mechanical signaling in stem cells is often simultaneously regulated by both “outside‐in” and “inside‐out” signaling, through interplays a complex interplay among ECM and cell membrane mechanics, mechanosensing, and intracellular signaling.^[^ ^23^ ^]^ Therefore, our focus is on using biomaterials for modulating the mechanical pathways of stem cells through “outside‐in” signaling. The crucial perspective of “inside‐out” signaling will also be introduced for a comprehensive understanding of the mechanical signaling network.

### 2.1 Cell Membrane and Cytoskeleton‐Mediated Mechanotransduction Pathways

Developing materials for the mechanomodulation of stem cell fates requires a fundamental understanding of key mechanosensing and mechanotransduction pathways. Therefore, we will first briefly introduce these pathways so biomaterial scientists can gain insights from cell biology and better tailor the biomaterials for regulating outside‐in signals.

The Hippo pathway, including YAP (Yes‐associated protein) and TAZ (transcriptional coactivator with PDZ‐binding motif), is perhaps the most studied effector for mechanical signaling. YAP and TAZ are transcriptional co‐activators that primarily act by binding to enhancer regions through the recruitment of transcriptional enhancer‐associated domain (TEAD) factors. They are mostly studied in the Hippo signaling pathways.^[^ ^24^ ^]^ Specifically, MST1 and MST2 (mammalian sterile 20‐like kinases 1 and 2) induce phosphorylation and activation of LATS1/LATS2 (large tumor suppressor 1/2) to further inactivate YAP/TAZ, which typically results in the retention and degradation of YAP/TAZ within cytoplasmic regions.^[^ ^25^ ^]^ Unlike these biochemical signaling cascades, mechanotransduction‐associated YAP/TAZ intracellular distribution has been discovered more recently and is independent of the previously identified kinases LATS1/LATS2.^[^ ^26^ ^]^ Instead, mechanotransduction mediated by YAP/TAZ is closely linked with cytoskeletal and actin dynamics.^[^ ^27^ ^]^ For instance, treatment of F‐actin inhibitors subsequently abolished mechanics‐induced nuclear relocation of YAP/TAZ.^[^ ^28^ ^]^ Nevertheless, the amount of F‐actin, or the ratio between F‐actin to G‐actin, does not correlate with YAP/TAZ cytoplasmic levels, while the F‐actin capping reagents (e.g., CAPZ and collin) seem to be the mediators between cytoskeletal dynamics and YAP/TAZ signaling.^[^ ^29^ ^]^

One of the unique aspects of YAP/TAZ pathways that is particularly important for mechanical signaling is their subcellular distribution‐dependent cascades. YAP/TAZ, as transcriptional co‐activators, are functional through gene regulation, which only occurs in the nucleus.^[^ ^30^ ^]^ Various ECM features, such as micro/nano topographies and stiffness, and external mechanical forces, such as shear forces, have been associated with specific patterns of YAP/TAZ intracellular distribution (cytoplasm vs. nucleus). For instance, in microenvironments characterized by low mechanical stimuli—such as soft extracellular matrices (ECMs) or reduced cell‐adhesive ligand density. These cells typically adopt a more rounded shape, which correlates with cytoplasmic rather than nuclear localization of YAP/TAZ. In contrast, stem cells develop stronger cytoskeletal tension in stiffer (elastic modulus greater than 10 kPa) or more adhesive ECMs, promoting nuclear localization of YAP/TAZ.^[^ ^31^, ^32^, ^33^ ^]^ Furthermore, more complicated mechanical cues subtly alter YAP/TAZ intracellular distribution. For example, lowering cell densities, switching from unidirectional to disturbed flow, and cell stretching could all lead to enhanced nuclear localization of YAP/TAZ.^[^ ^34^, ^35^ ^]^ Additionally, although a stiffer matrix promotes nuclear localization of YAP/TAZ in both 2D and 3D cell cultures, the 3D environment leads to more heterogeneous spatial patterns of YAP/TAZ distribution, a feature critical for accurately recapitulating complex tissue organization in organoid cultures.^[^ ^36^, ^37^, ^38^ ^]^

Importantly, the alteration of the intracellular distribution of YAP/TAZ elicited by varying mechanics of ECM and surrounding cells directly leads to changes in gene expression patterns that critically regulate various types of stem cell fates. In general, accumulation of YAP/TAZ in the nucleus versus cytoplasm has been correlated with more proliferative populations of adult stem cells,^[^ ^39^, ^40^ ^]^ faster self‐renewal of ESCs,^[^ ^41^, ^42^ ^]^ enhanced osteogenesis from MSCs,^[^ ^43^ ^]^ cardiomyogenesis of cardiomyocyte progenitor cells,^[^ ^44^, ^45^ ^]^ and faster cell migration in wound healing.^[^ ^46^ ^]^ Therefore, biomaterials that exert specific forces on the cell surface and alter YAP/TAZ intracellular distribution could ultimately regulate stem cell fates. It is thus crucial to achieve reproducible and predictable external mechanical stimuli, YAP/TAZ distribution, and downstream gene expression and stem cell fate regulation for future mechanotherapies.

As key mediators of mechanotransduction, Piezo channels play a critical role in controlling stem cell fate by sensing and responding to mechanical stimuli in the cellular microenvironment. Piezos (including Piezo1 and Piezo2 for vertebrates) are non‐specific ion channels widely existent in different types of human cells that can be activated by mechanical forces such as shear flow.^[^ ^47^ ^]^ However, the molecular mechanism of Piezo activation remains unclear, with two dominant theories in the field, known as the ‱force‐from‐lipids^[^ ^48^, ^49^ ^]^ and “force‐from‐filaments” model.^[^ ^50^, ^51^ ^]^ Still, the consequences of the activation of Piezos have been extensively studied, concluding that changes in membrane configuration or stiffness induced by external stimuli can result in the activation or deactivation of Piezo activities with altered mechanosensitivities.^[^ ^52^, ^53^ ^]^ For instance, decreased membrane stiffness sensitizes cells toward membrane tension and Piezo activation under the same extracellular forces.^[^ ^54^ ^]^ As such, inhibitors of actin, dynamin, and filamin A have all been found to lower membrane stiffness and thus lower Piezo 1 activity.^[^ ^55^ ^]^ In contrast, cells undergoing osmosis would have increased Piezo activities due to a higher cell membrane tension.^[^ ^56^, ^57^ ^]^

Considering the pivotal role of membrane mechanics in regulating Piezo activity, mechanical stretching of cells frequently induces Piezo‐mediated signaling pathways.^[^ ^52^ ^]^ Among the downstream mechanoresponses of Piezo activation, the extracellular signal‐regulated kinase (ERK) pathway is the most significant and associated with various stem cell fate determinations. Another mechanism is the calcium influx associated with actin polymerization dynamics that alter cytoskeleton contractility and cell mechanics, which in turn leads to activation/deactivation of downstream serum response factor (SRF).^[^ ^58^, ^59^ ^]^ Nevertheless, the alteration of Piezo mechanosensitivity by other mechanical properties of ECM remains to be elucidated. So far, activation of Piezo has been found to play a crucial role in cardiovascular tissue maturation. For example, shear stress during embryogenesis triggers the migration and alignment of endothelial cells for vascular formation.^[^ ^60^ ^]^

Besides YAP/TAZ and Piezo, there are other mechanosensitive pathways, including transforming growth factor‐β (TGFβ) families, Wnt/β‐catenin, Notch, and fibroblast growth factor (FGF) pathways, which often participate in the coordination of mechanical signaling for guiding stem cell fate decisions.^[^ ^61^, ^62^, ^63^, ^64^, ^65^, ^66^ ^]^ However, the details on how mechanical forces activate these pathways and signal downstream cascades remain largely unknown. Understanding these pathways and discovering other mechanosensitive proteins are both fundamentally important in mechanobiology and essential for designing better biomaterials to present desired mechanical cues to stem cells in tissue engineering.

### 2.2 Nuclear Membrane‐Mediated Mechanotransduction Pathways

External mechanical forces can not only induce direct biochemical alterations of protein localization, deactivation, and activation but also affect the shape and mechanics of the nucleus, which leads to epigenetic changes in stem cell fate decisions.^[^ ^67^, ^68^ ^]^ The force transmission process to the nucleus and alteration of cell gene expression is defined as nuclear mechanotransduction.^[^ ^69^ ^]^ The linker of nucleoskeleton and cytoskeleton (LINC) and nuclear envelop transmembrane (NET) proteins are key force mediators for nuclear mechanotransduction.^[^ ^70^, ^71^ ^]^ Specifically, like YAP/TAZ‐mediated mechanotransduction, nuclear mechanotransduction starts when forces are transmitted by transmembrane receptors (e.g., integrins, ion channels such as Piezos) or direct alterations on cell surface mechanics. This process either directly propagates mechanical signals to the nucleus via the cytoskeleton or induces biochemical changes, such as actin polymerization, that alter cytoskeletal dynamics.^[^ ^72^ ^]^ Through the LINC and NET proteins, forces transmitted from the cytoskeleton to the nucleoskeleton can induce two major changes: i) the apico‐basal polarity and structure organization of nuclear basal lamina; and ii) the nuclear pore structure and molecular trafficking dynamics across the pore.^[^ ^73^, ^74^ ^]^

Unlike YAP/TAZ‐mediated mechanotransduction, nuclear mechanotransduction is highly tissue‐specific, as it involves the expression, activation, and suppression of several different genes in a less predictable manner, dependent on 3D conformational changes in nuclear organization and nuclear pores induced by mechanical forces.^[^ ^67^ ^]^ Studies have demonstrated that cells respond to increased ECM stiffness and mechanical load by upregulating lamin A/C expression and altering the lamin A/C:B ratio. This response may be mediated by changes in lamin A/C assembly dynamics, with phosphorylation playing a key role in regulating these processes.^[^ ^75^ ^]^ Similarly, high mechanical force applied on nesprin‐1 in the LINC/NET protein family can stiffen the nuclear envelope and induce more emerin phosphorylation and assembly of lamin A.^[^ ^76^ ^]^ However, when stem cells are exposed to extreme mechanical forces, LINC could disassemble, and the level of response significantly vary depending on cell types with different Sad1 Unc‐84 Domain Protein 1 (SUN1): Sad1 Unc‐84 Domain Protein 2 (SUN2) ratio and lamin A levels.^[^ ^77^ ^]^ MSCs exposed to cyclic tensile strain showed rapid SUN2 turnover (in a few minutes), increased SUN1:SUN2 ratio, and reduced nuclear coupling with cytoskeleton.^[^ ^78^ ^]^ Therefore, cells at lower endogenous SUN2 levels would be less sensitive to strain‐induced nuclear changes. Similarly, cells with more lamin A would show more heterochromatin softening in response to cyclic stretch. However, nuclear mechanotransduction may involve more intricate, cell‐ and tissue‐specific pathways, necessitating substantial research efforts to fully elucidate this critical phenomenon.

LINC serves not only as a mechanical force transmitter from the cytoskeleton to the nucleus, but also regulates gene expression by mediating key biochemical signals for controlling stem cell differentiation. Studies have suggested that force‐induced β‐catenin activation could be mediated by phosphorylation of nesprins and SUN in the LINC, which can directly promote β‐catenin importation into the nucleus.^[^ ^79^ ^]^ Meanwhile, emerin in the intranuclear region of LINC can bind to and deactivate β‐catenin by exportation across nuclear pores.^[^ ^80^, ^81^ ^]^ Therefore, LINC plays a crucial role in determining the cellular mechanosensitivity associated with force‐induced activation/deactivation of β‐catenin pathways. Another relevant example is that the assembly of monomeric actin at the perinuclear region can deplete the actin pool within the nucleus, which induces changes in chromatin remodeling complexes and RNA polymerase activity, thus affecting gene expression. For example, the myocardin‐related transcription factor A (MRTF‐A) signaling has been closely regulated by the actin dynamics and thus the nuclear envelope‐mediated biomechanical pathways.^[^ ^82^ ^]^ Therefore, LINC, including its composition, assembly, stoichiometry, and localization, closely regulates the nuclear mechanotransduction, and variations in LINC are largely responsible for the heterogeneous responses toward mechanical cues in different cell and tissue types.

### 2.3 Epigenetic Modulation‐Mediated Mechanotransduction Pathways

Force transmitted to the nucleus would eventually induce changes in gene expression patterns for regulating stem cell differentiation. Epigenetic modifications, such as histone methylation and acetylation that regulate nuclear gene expression patterns, are fundamental in determining cellular functions and guiding organogenesis.^[^ ^83^ ^]^ Forces transmitted to the nucleoskeleton induce conformational changes in chromatin and the accessibility of genes at different regions in the nucleus. That said, one can imagine that the 3D organization of the genome within the nucleus is highly variable under different force strengths, directions, and dynamics. In general, the genome could be categorized into active, repressed compartments, as well as regions with probabilistic radial positioning of activated domains that are highly dependent on the original epigenetic status of the cell.^[^ ^84^ ^]^ To the best of our knowledge, there has been no well‐established model for predicting epigenetic changes when cells are exposed to specific force patterns. However, studies suggest that the nuclear periphery and regions surrounding the nucleolus tend to contain more silenced genes. In contrast, the central ring contains more activated genes, which is demonstrated by an advanced single‐cell Hi‐C technique.^[^ ^85^ ^]^ This unique nuclear periphery distance‐dependent regulation of gene expression has been partially attributed to lamina‐associated domains (LADs) in addition to genomic organization, which are verified in both neurogenesis and adipogenesis processes.^[^ ^84^ ^]^

**Figure 3:**
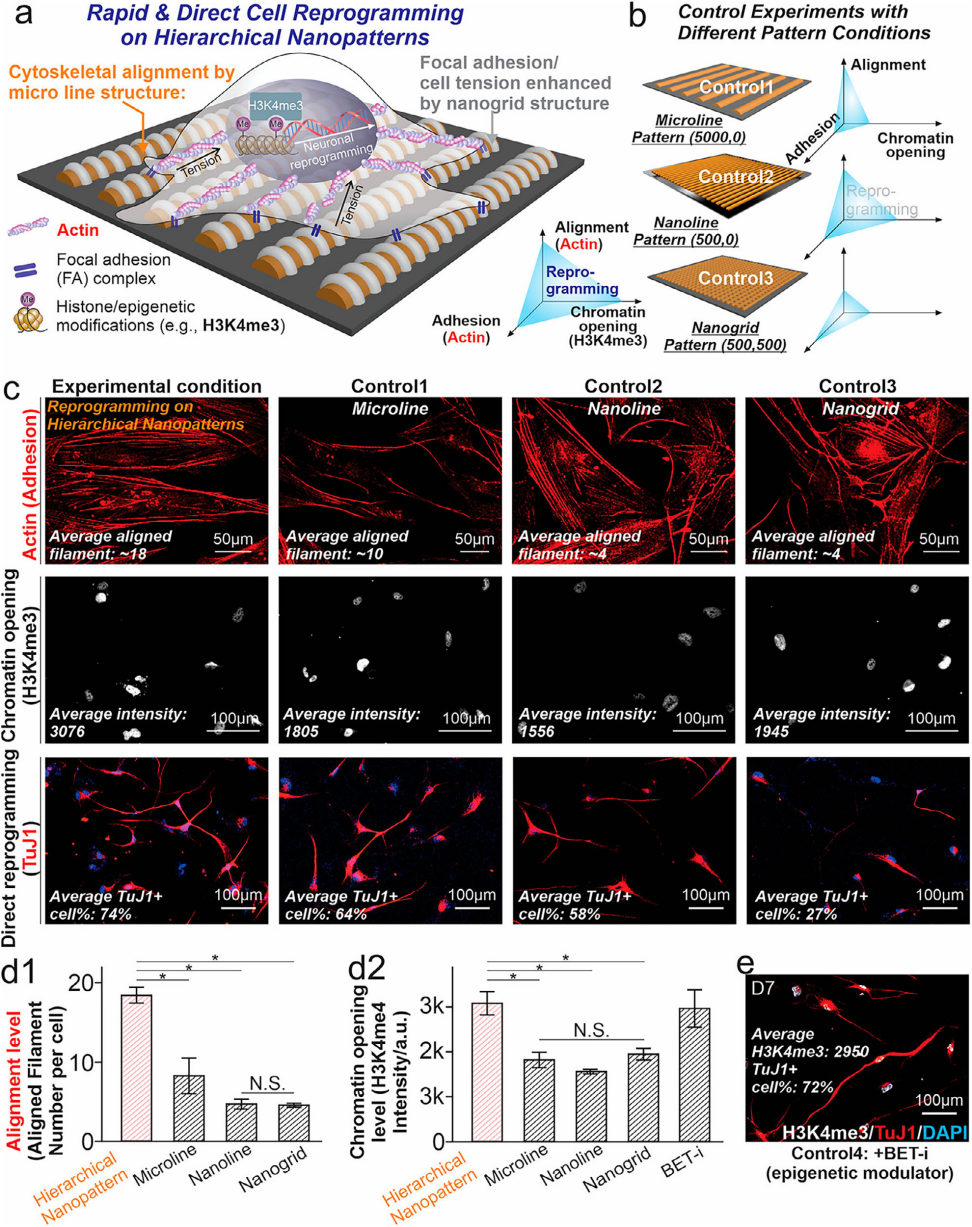
Nuclear mechanotransduction for inducing cell neuronal reprogramming by nanotopography. a,b) Schematic illustration of the proposed mechanism underlying enhanced direct neuronal reprogramming on hierarchical nanopatterns (a) compared to conventional micro/nanopatterned substrates (b). Micropatterned lines promote the alignment of cytoskeletal filaments, while nanoscale features enhance focal adhesion formation and mechanical tension in the aligned fibers. Together, these biophysical cues synergistically facilitate chromatin relaxation and help overcome epigenetic barriers to cellular reprogramming. c,d) Immunostaining (c) and quantitative analysis (d) of cytoskeletal organization and chromatin accessibility (marked by H3K4me3) in fibroblasts demonstrate increased alignment of actin filaments and elevated levels of epigenetic modification to Histone H3 in cells cultured on hierarchical nanopatterns compared to control surfaces. These effects contribute to significantly improved neuronal reprogramming efficiency. Data represent *n* = 3 biological replicates; *P < 0.05 by one‐way ANOVA with Tukey's post hoc test. e) Representative immunofluorescence image showing rapid and efficient neuronal reprogramming of fibroblasts cultured on a control substrate following treatment with an epigenetic modulator (BET inhibitor, BET‐i). Reproduced with permission.^[^ ^86^ ^]^ Copyright 2022, NatureACS Nano.

Biomaterials that present mechanical cues to cells have also been found to alter the epigenetic status of somatic and stem cells to instruct their fate decisions (**Figure** **3**).^[^ ^86^ ^]^ Subtle changes in material properties—such as cell adhesion ligand density, stiffness, viscoelasticity, shape, compression, and tension—have been associated with various epigenetic modifications. However, the precise mechanical signaling mechanisms involved are often overlooked. Among various types of histone modifications responsible for epigenetic regulation of cell fates, several are among the most commonly reported in stem cell differentiation and cellular reprogramming: histone H3 acetylation (H3ac), histone H3 lysine 4 trimethylation (H3K4me3)‐based regiospecific gene activation, and trimethylation of lysine 27 on histone 3 (H3K27me3)‐based regiospecific gene repression. Specifically, microgroove patterns are reported to reduce histone deacetylase (HDAC) activities for reprogramming somatic cells into iPSCs.^[^ ^87^ ^]^ Culturing human epithelial cells cultured on 3D laminin‐rich ECM leads to round‐shaped cells with condensed chromatin status.^[^ ^88^ ^]^ However, so far, there has been a lack of systematic understanding and predictive models on how different biomaterial properties could modulate specific chromatin organization that results in specific stem cell fate control.^[^ ^89^ ^]^ Expanding the library of biomaterials for mechanomodulation and advancing materiomics methodologies could be essential strategies for bridging this knowledge gap.

Another example of epigenetic modulation‐mediated mechanotransduction is nuclear deformation and alteration of nuclear pore‐mediated transportation, which further influences cellular reprogramming through epigenetic modulation. Mouse fibroblasts underwent 5 µm microchannel deformation and were then cultured in media with classical neuronal reprogramming BAM (BRN2, ASCL1, and MYT1L) factor cocktail expressed by doxycycline (Dox)‐controlled viral vectors for 7 days. It was then found that, compared to cells flowed through the control (200 µm wide) microfluidic device, cells treated by 5 µm microchannels 6 h prior to the Dox induction showed a significant neuronal reprogramming outcome (over six‐fold increase in neural marker TuJ1 positive cells).^[^ ^68^ ^]^ To confirm that neuronal reprogramming is truly enhanced by transient nuclear deformation induced within the microchannels, epigenetic states were first analyzed by a Förster resonance energy transfer (FRET)‐based biosensor that reports the activities of histone H3 lysine 9 trimethylation (H3K9me3). The increase in the H3K9me3 signals confirmed that nuclear deformation leads to epigenetic changes associated with neuronal reprogramming during the flow through the microchannels. Furthermore, euchromatin and heterochromatin staining after prolonged culture of cells squeezed by the microfluidic channel show that the epigenetic modulation by transient nuclear deformation has continuous effects even after the mechanomodulation. In contrast, histone acetylation markers, including AcH3 and H3K9ac, did not show significant differences compared to control groups. However, 5‐methylcytosine (5‐mC) levels were notably reduced in the 5 µm microchannel‐treated group relative to controls. Furthermore, treatment with Bix01294, a H3K9me3‐specific inhibitor, negated the observed enhancement in neuronal reprogramming. As one possible mechanism linking nuclear deformation with epigenetic changes, Lamin A/C shifts from the nuclear membrane into the nucleoplasm, while Lamin B maintains a constant position, indicating the major role of Lamin A/C in enhancing neuronal cellular reprogramming. Indeed, silencing Lamin A/C abolished the enhancement in terms of cellular reprogramming. Taken together, these results collectively support that the nuclear deformation induced by mechanical squeezing leads to prolonged effects on epigenetics and neuronal reprogramming.^[^ ^68^ ^]^

## 3 Mechanobiological Dynamics in Stem Cell Fate Regulation

### 3.1 Mechanobiological Dynamics of Natural Extracellular Matrix

Stem cells constantly interact with ECM dynamically, with both outside‐in and inside‐out signals influencing cellular fate.^[^ ^18^, ^90^ ^]^ ECM dynamics can be described by its changes in composition, structure, surface ligands, cross‐linking, and physical properties (such as stiffness, viscoelasticity, and others).^[^ ^91^ ^]^ Natural ECM is composed of collagen of different types, laminin, glycosaminoglycans (e.g., hyaluronic acid and chondroitin sulfate), fibronectin, tenascin, and other biomolecules.^[^ ^92^ ^]^ ECM is usually stable but degrades or dissolves in response to stimuli such as pH, proteases, enzymes, and reactive oxygen species (ROS).^[^ ^93^, ^94^ ^]^ Additionally, ECM‐modifying molecules, including lysyl oxidases, lysyl hydroxylases, and transglutaminases, can reinforce the stability and cross‐linking of ECM via compositional changes.^[^ ^95^ ^]^ The dynamics of extracellular matrix (ECM) compositional changes, such as degradation and cross‐linking, are primarily governed by enzymatic activity and diffusion processes, with characteristic half‐times on the order of hours. These timescales are biologically significant because they align with the pace of tissue remodeling and repair, while also influencing the progression of pathological processes such as fibrosis, cancer invasion, and inflammation.^[^ ^96^ ^]^ Alterations in pH induce rapid hydration or dehydration of the extracellular matrix (ECM), resulting in significant changes to its mechanical and structural properties within seconds to minutes. However, the change in pH typically takes time, with inflammation‐induced pH change occurring in hours and days, while the injury‐induced pH change occurs rapidly due to massive apoptosis of cells at its acute phases.

The mechanical dynamics of ECM can be even more complex, and it has recently become the frontier of tissue engineering research.^[^ ^97^ ^]^ ECM stiffness, vital for cell fate control and disease progression, was identified three decades ago, and our understanding of its role has undergone dramatic changes in different contexts. Blood vessels experiencing pulsatile blood flow show distensible properties to accommodate the forces, but become more elastic with increased stiffness when undergoing fast continuous flow to prevent vessel rupture. Mechanical dynamics of cells and tissues can be highly complex. The brain is a highly dissipative viscoelastic organ, exhibiting elastic‐like properties when forces are applied at millisecond and mega‐pascal scales, but more like glass and liquid when forces are applied for weeks‐ and at tens of pascal scales. Different regions of the brain can be distinguished by their viscoelastic properties. These properties are common to most human tissues, which exhibit elasticity at high frequencies (1 Hz or above) and large stresses, but behave with greater viscosity at low frequencies and small stresses (<20% of their storage moduli).^[^ ^98^, ^99^, ^100^ ^]^ Soft tissues, including the brain, adipose tissues, breast, liver, muscle, and skin, relax their resistance to deformation more substantially across tens to hundreds of seconds. Storage modulus‐loss modulus plot at 1 Hz frequency and time‐normalized stress plot show the highly diverse viscoelastic properties of different types of ECM, soft tissues, and stiff tissues.^[^ ^101^ ^]^ Additionally, even within the same tissue, various factors—including different regions, times of development, or disease progression—can result in significant differences in viscoelasticity. Some factors (e.g., viscoelasticity changes in breast cancer development) have been established as a disease hallmark.

### 3.2 Mechanobiological Dynamics at Cell Membranes

Small molecules within the plasma membrane can interact with the ECM, enabling bidirectional transmission of mechanical forces between the ECM and the cytoskeleton.^[^ ^21^, ^102^, ^103^ ^]^ In the classical fluid‐mosaic model, bilayer membranes are classified into detergent‐resistant and detergent‐labile domains, each containing distinct sets of components such as transmembrane proteins, glycosylphosphatidylinositol (GPI)‐anchored proteins, lipidated proteins, and, more recently discovered, lipid‐associated RNAs, alongside varying compositions of unsaturated and saturated lipids in the outer membrane.^[^ ^104^, ^105^ ^]^ Detergent‐resistant and detergent‐labile membrane components partition into sub‐micrometer domains. Lipid raft theory posits that cooperative interactions among cholesterol, saturated lipid species, and glycosylated lipids generate the thermodynamic basis for domain self‐assembly. These molecular interactions drive the dynamic formation and functional specialization of lipid rafts as membrane‐organizing centers.^[^ ^21^, ^106^, ^107^, ^108^, ^109^ ^]^ Mammalian cells can generate over 100 different membrane lipids, and these lipids are extremely dynamic, constantly remodeled by changes in cell condition, metabolism, and microenvironment, including ECM. At the biomolecule level, cell membranes show a fluid‐like property with lipids and proteins interacting with each other via diffusion. Plasma membranes transmit mechanical forces between the ECM and the cytoskeleton through both biophysical mechanisms (such as membrane bending) and biochemical mechanisms (such as cellular adhesion).^[^ ^110^ ^]^ In the lipid rafts, transmembrane proteins play essential roles in regulating both biophysical and biochemical mechanisms and strongly influence cell metabolism, transport, and signaling.^[^ ^111^ ^]^ Transmembrane proteins comprise 30% of total cellular proteins and 60% of all drug targets.^[^ ^112^ ^]^ These proteins constantly interact with the surrounding lipids via their lipophilic domains and the ECM and signaling molecules via transporters, channels, and signal receptors. At the lipid raft scale (tens to hundreds of nanometers), membrane molecules begin to form stable lipid bilayer structures and self‐organize into distinctive functional domains.

At the micrometer scale, membranes can be viewed as robust, elastic 2D sheets capable of stretching, bending, and adopting diverse shapes in response to mechanical and biochemical signals from the cytoplasm and ECM. The dynamics of protein‐lipid interactions are governed by a variety of rules. For example, transmembrane domains (TMDs) of proteins prefer to insert into bilayers with similar hydrophobic thickness, and thus, hydrophobic mismatch in modified membrane proteins can cause exclusion from the membrane or clustering of such TMDs to match the hydrophobic thickness.^[^ ^113^ ^]^ Additionally, lipid‐protein interactions can induce conformational changes in proteins, as observed in channel‐gating proteins like Piezo. On the other hand, biochemical induction of conformational changes of proteins can also induce changes in the lipid conformation.^[^ ^114^ ^]^ Rapid cellular processes, such as protein incorporation into lipid membranes, structural changes due to external stimuli, and channel opening, often occur within milliseconds. Protein synthesis, by contrast, is considerably slower, taking minutes to complete. In a Dictyostelium cell model engineered with a fluorescently labeled PIP3 biosensor, biochemical activation of Ras/PI3K/Akt/F‐actin pathways induces depletion of membrane proteins at the scale of tens of seconds by using dynamic partitioning as a key mechanism, highlighting the dynamic properties of lipid membranes.^[^ ^115^, ^116^, ^117^ ^]^

Focal adhesion formation is another crucial biochemical process occurring during the force transmission from the ECM to the cytoskeleton, mediated by the membrane, but at a much slower dynamics as compared to biophysically induced change of membrane curvature and membrane protein reconfiguration.^[^ ^118^ ^]^ Using an optical tweezer, the dynamics of integrin adhesion complex formation have been studied using fibronectin‐coated microbeads localized at the leading edge of a focal adhesion.^[^ ^119^ ^]^ From this study, it was found that the initial interaction between ECM and cell membrane can be formed rapidly but at a weak adhesion force (<5 pN). This weak adhesion can be reinforced by cytoskeletal interactions when the cell detects local resistance. This reinforcement can occur within 10 seconds, and the adhesion force can reach 60 pN. As such, the mechanosensing protein plays a vital role in the mechanotransduction and membrane dynamics. There are over 50 molecules involved in the formation, stabilization, and mechanosensing of focal adhesion complexes. These molecules include the integrins with ECM‐binding α and β subunits, as well as non‐integrin components such as adhesive molecules (e.g., syndecan‐4, layilin, uPAR, and caveolin) and signaling molecules (e.g., PtdIn(4,5)P2) that regulate cytoskeleton‐cell membrane interaction and actin polymerization.^[^ ^120^ ^]^ In addition to these external domains, there are also inner membrane‐bound domains, including talin, filamin, tensin, and α‐actinin, which link integrin, membrane, and cytoskeleton. Other sub‐membrane plaque‐associated molecules and signalling molecules (e.g., focal adhesion kinase, integrin‐linked kinase) influence focal adhesion via indirect effects on the cytoskeleton. Their responses to external forces vary significantly and have not been well understood in terms of the detailed dynamics.^[^ ^121^, ^122^ ^]^ However, most biochemical processes involved in supporting focal adhesion formation typically occur on a timescale of seconds.

Given the crucial roles of cell membranes in mechanotransduction, there has been a great interest in developing practical modulation approaches to regulate cell membrane functions both mechanically and biologically. First, there are receptors on the cell membranes that can directly respond to changes in membrane tension to mediate the downstream signalling. One of the most classical mechanical sensors is the Piezo channel, which opens under tension, stretching, and shear flow to induce the calcium influx. For example, Gudipaty et al. found that activation of Piezo1 by mechanical stretching leads to rapid division of epithelial cells.^[^ ^123^ ^]^ In low‐density regions where cells are stretched, Piezo1 is localized to the plasma membrane and cytoplasm, enabling it to sense mechanical stretch. This activation stimulates calcium influx, which triggers phosphorylation of ERK1/2 and upregulation of cyclin B, pushing cells paused in early G2 into mitosis. In contrast, in crowded regions, Piezo1 accumulates in cytoplasmic aggregates and responds to mechanical compression by initiating cell extrusion, leading to apoptosis. This dual function ensures a balance between cell proliferation and loss, preventing barrier disruption and tumorigenesis. The spatial localization and mechanical context of Piezo1 activation ultimately determine whether epithelial cells divide or are extruded, tightly regulating tissue homeostasis. On the other hand, Piezo 1 also plays a role in determining the mechanical properties of cells. Using traction force microscopy, Pang et al. demonstrate that blocking Piezo1 and the downstream transcription factor Grhl3 can increase the traction force generated by cytotoxic T cells, which potentiates tumor immunotherapy. Based on a similar strategy, Wang et al. have further engineered T cells to selectively express a chimeric antigen receptor (CAR) in response to ultrasound, which triggers Piezo1 activation and calcium influx to activate the genetically engineered NFAT transcription factor as a promoter for CAR.^[^ ^124^ ^]^ This interplay between the Piezo1 channel and the mechanical states of cell membranes highlights the complexity of receptor‐mediated mechanotransduction.

**Figure 4:**
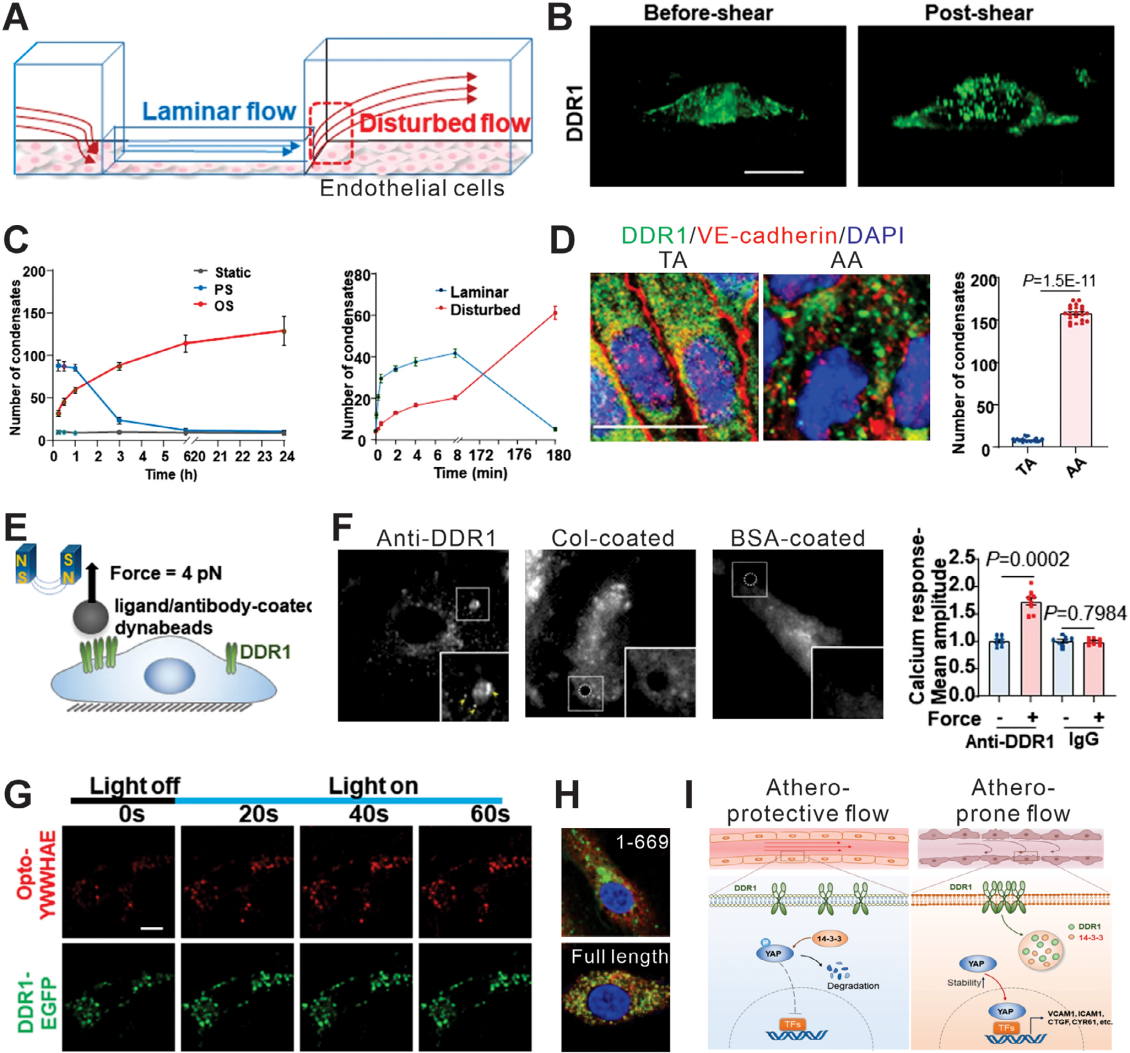
DDR1 as a novel mechanosensory protein for mediating flow‐induced mechanotransduction in endothelial cells. A) Schematic diagram illustrating a 3D microfluidic model representing different vascular flows used for the study of DDR1 mechanotransduction. B) Confocal images of DDR1 droplet formation in HUVECs after undergoing laminar flow for 2 min. C) Quantification of DDR1 condensates in HUVECs after pulsatile shear or laminar shear (PS or LS) or oscillatory shear (OS) over varying time intervals. D) Immunofluorescent staining of DDR1 (green) and VE‐cadherin (red) in the aortic arch (AA) and thoracic aorta (TA) of C57BL/6 wild‐type mice. Quantification of DDR1 condensates is shown in the graph on the right. *n* = 20 images obtained from 5 biological replicates of mice. E) Illustration of the magnetic tweezer‐based manipulation of the DDR1 receptor. F) Total internal reflection fluorescence (TIRF) microscopy imaging showing DDR1 condensate formation in DDR1 adenovirus‐infected HUVECs. The graph on the right shows quantification of the DDR1 condensate fluorescent signals. G) Confocal microscopy images showing NIH3T3 cells with an engineered fluorescent gene reporter on Opto‐YWHAE and DDR1‐EGFP. Scale bar, 10 µm. H) Live cell fluorescent imaging of DDR1 with 1–669 aa, 1–693 aa, DDR1‐FL, and YWHAE‐Cherry in engineered EA.hy926 ECs. Cells were subjected to flow for 3 h before the imaging. I) Summary of the DDR1‐mediated mechanotransduction. DDR1 acts as a key mechanosensor in endothelial cells, regulating YAP activity in response to shear flow. Under laminar and atheroprotective flow, YAP is phosphorylated, binds to 14‐3‐3 proteins, and is retained in the cytoplasm for degradation. In contrast, under disturbed, atheroprone flow, DDR1 senses mechanical stress and undergoes force‐induced oligomerization, forming liquid‐like condensates with 14‐3‐3. These condensates inhibit YAP phosphorylation, allowing its nuclear translocation and activation, which contributes to endothelial dysfunction. Figures reproduced with permission.^[^ ^125^ ^]^ Copyright 2023, Nature.

In addition to Piezo and other conventional mechanical sensors, novel membrane proteins have recently been discovered, shedding light on their roles in mechanosensing. For instance, discoidin domain receptor one (DDR1) is previously known in terms of its role as a tyrosine kinase (**Figure** **4**). By observing a flow‐induced activation of DDR1 in endothelial cells, Zhou et al. establish DDR1 as a novel mechanosensory membrane protein. Specifically, shear force could induce conformational changes in the DDR1 ectodomain to expose the buried cysteine‐287.^[^ ^125^ ^]^ The exposure of cysteine‐287 then promotes receptor oligomerization and phase separation in response to mechanical stresses. Interestingly, shear force could induce the formation of DDR1 liquid‐like biomolecular condensates, which incorporate YWHAE that facilitates the nuclear translocation of YAP. Similarly, Xu et al. recently verified the novel role of the NINJ1 protein in maintaining the mechanical robustness of the cell membrane.^[^ ^126^ ^]^ NINJ1 is previously known for its role in pyroptosis and lytic cell death. However, through combined mechanobiological screening, NINJ1 was found to be inversely correlated with forces required for membrane rupture. As such, mechanobiological dynamics at cell membranes play increasing roles in regulating cell mechanotransduction, and novel functions of membrane proteins in mechanosensing are expected to be revealed in the future.

### 3.3 Mechanobiological Dynamics at the Cytoskeleton

The cytoskeleton is mainly composed of actin filaments, microtubules, and intermediate filaments. The cytoskeleton senses and transmits forces from cell membranes to the nucleus via the dynamic polymerization and depolymerization of the filament network in the cytoplasm. When focal adhesion initiates on the cell membrane, actin polymerizes at the leading edge (e.g., lamellipodia or filopodia) within seconds, a process critically regulated by Rac1/Cdc42.^[^ ^127^ ^]^ As focal adhesion becomes more mature, vinculin, talin, and paxillin are recruited within minutes which stabilizes the mechanical linkage between ECM and actin stress fibers.^[^ ^128^ ^]^ Afterward, RhoA–ROCK–myosin II signaling evolves at the timescale of tens of minutes, which is dependent on substrate stiffness.^[^ ^129^ ^]^ Around 1–2 h later, the cytoskeleton reaches steady state and completes the adhesion process. YAP/TAZ signaling occurs at sites of actin filaments, and the nuclear translocation will occur hours after the stable adhesion forms.^[^ ^130^ ^]^ In addition to actin filaments, microtubules play a crucial role in modulating mechanotransduction processes; however, they are generally less dynamic than actin, with polymerization and depolymerization occurring over timescales ranging from seconds to minutes. Mechanically induced microtubule stabilization occurs when there is shear stress or compression. Under sustained mechanical load, microtubule stabilization can be observed within 10–30 min.^[^ ^131^ ^]^ Microtubule inhibition by nocodazole can increase actomyosin contractility within 5–10 min, partially due to alteration of RhoA signaling. Microtubules also play a logistical role in trafficking proteins, vesicles, and signaling complexes to sites of mechanical activity. This targeting process, including delivery to focal adhesions or membrane protrusions, typically occurs on the 10–30 min scale and is essential for long‐term adaptation to mechanical signals. Compared to actin and microtubules, intermediate filaments (e.g., vimentin and keratins) show slower remodeling dynamics in response to mechanical signals. However, it provides more sustained tension support from minutes to hours. Specifically, vimentin exhibits turnover rates ranging from 30 to 120 min under homeostatic conditions, which slightly accelerate during force‐induced remodeling.^[^ ^132^ ^]^ Upon mechanical stress (e.g., stretching or compression), vimentin networks gradually reorganize within 15–30 min, redistributing tension across the cytoplasm. In long‐term stress (e.g., stiff ECM or high traction), intermediate filaments reinforce and bundle to resist forces, which contributes to mechanical memory. Nuclear lamins, which link cytoskeletal tension to nuclear deformation via the linker of nucleoskeleton and cytoskeleton LINC complex, show slower turnover rates, with half‐lives of hours to days. However, force‐induced chromatin reorganization and nuclear softening can occur within 30–60 min, establishing a temporal link between cytoskeletal tension and transcriptional regulation.^[^ ^133^ ^]^

### 3.4 Mechanobiological Dynamics at the Nucleus

External mechanical forces can profoundly impact cellular behavior by inducing biochemical alterations and reshaping the nucleus, leading to epigenetic changes that influence stem cell fate decisions. This process, known as nuclear mechanotransduction, refers to the transmission of mechanical signals from the ECM and cytoskeleton to the nucleoskeleton, resulting in changes in gene expression and cellular functions.^[^ ^69^, ^134^ ^]^ At the core of this process are the LINC complex and nuclear envelope transmembrane proteins (NET), which serve as key mediators, linking the cytoskeleton to the nucleoskeleton. When external mechanical forces are transmitted through transmembrane receptors, such as integrins or ion channels like Piezos, the mechanical signal is either directly propagated through the cytoskeleton or results in biochemical changes like actin polymerization, which in turn alter cytoskeletal dynamics. These changes are then communicated to the nucleus via the LINC and NET proteins, which can affect the nuclear envelope, nuclear pore structure, and gene accessibility.^[^ ^67^ ^]^ Mechanical forces induce structural changes in the nucleus, including alterations in nuclear basal lamina organization and nuclear polarity, as well as changes in chromatin accessibility that impact gene expression.^[^ ^87^, ^135^ ^]^ These nuclear responses are dependent on the force's magnitude, direction, and frequency, with mechanical signals initiating rapid events (within milliseconds to minutes) through integrin clustering and cytoskeletal reorganization, followed by structural nuclear changes on a timescale of minutes to hours.^[^ ^136^ ^]^ For instance, forces exerted on the LINC complex cause nesprin‐1 stretching, subsequently triggering emerin phosphorylation and lamin A assembly, typically occurring within minutes.

The mechanical signals also affect the assembly dynamics of lamin A/C, which can modulate chromatin organization and gene expression.^[^ ^137^ ^]^ Interestingly, nuclear mechanotransduction is tissue‐specific, as the response to mechanical forces can vary significantly between cell types, influenced by the expression levels of proteins like SUN1/2 and lamin A.^[^ ^138^ ^]^ For example, mesenchymal stem cells (MSCs) exposed to cyclic tensile strain exhibit rapid turnover of SUN2 within minutes and alterations in the SUN1/SUN2 ratio, which leads to changes in nuclear coupling with the cytoskeleton. This effect is more pronounced in cells with higher levels of lamin A, where heterochromatin softening occurs more rapidly. Additionally, force‐induced activation of β‐catenin in the LINC complex plays a significant role in gene regulation, as force triggers the phosphorylation of nesprins and SUN proteins, facilitating the importation of β‐catenin into the nucleus, while emerin can bind and export β‐catenin to deactivate it. Other examples include the depletion of actin from the perinuclear region, which induces chromatin remodeling and regulates RNA polymerase activity, affecting gene expression. Forces can also alter epigenetic modifications like histone acetylation (H3ac) and methylation (H3K4me3 and H3K27me3), key players in regulating gene expression during stem cell differentiation and cellular reprogramming.^[^ ^87^ ^]^ The timescales for these epigenetic changes range from minutes to hours, with the effects on histone modifications dependent on the strength, frequency, and direction of the applied mechanical forces. Studies have shown that biomaterials that present mechanical cues to cells, such as changes in stiffness, adhesion ligand density, and viscoelasticity, can alter epigenetic landscapes, influencing chromatin organization and gene expression.^[^ ^139^, ^140^ ^]^ For instance, microgrooves in materials can affect histone deacetylase (HDAC) activity, which is critical for reprogramming somatic cells into induced pluripotent stem cells (iPSCs). Furthermore, exposure to 3D ECMs, particularly those enriched in laminin, leads to changes in chromatin status and cellular morphology, affecting gene expression. However, while the effect of mechanical cues on epigenetics is well‐documented, the precise molecular dynamics and timescales remain complex and are still not fully understood. Overall, the process of nuclear mechanotransduction is governed by intricate molecular events, where LINC complex dynamics, nuclear envelope remodeling, and cytoskeletal reorganization occur over timescales ranging from milliseconds to hours, ultimately driving changes in gene expression, chromatin accessibility, and epigenetic regulation. The mechanotransduction pathways are highly cell‐ and tissue‐specific, with different responses to mechanical forces observed based on the levels of key proteins like lamin A and SUN proteins. The continued exploration of the molecular and temporal dynamics of mechanotransduction, particularly through biomaterial‐driven mechanical cues, will likely enhance our understanding of stem cell fate and regenerative medicine. Further research into these mechanical signaling pathways and their biomechanical regulation will be critical in developing more effective strategies for manipulating stem cell differentiation and harnessing mechanical forces to guide cellular processes.

### 3.5 Mechanobiological Dynamics at Other Cell Organelles

Effects of mechanotransduction on other cell organelles, such as mitochondria, endoplasmic reticulum, and Golgi apparatus, have not received sufficient attention until recently.^[^ ^141^, ^142^ ^]^ Mechanical stimuli, such as cyclic stretching or ECM stiffening, were initially found to modulate mitochondrial membrane potential and ATP production on the timescale of minutes. Later, mechanically induced cytoskeletal tension was found to enhance DRP1 recruitment to mitochondrial fission sites and promote fission for hours.^[^ ^143^ ^]^ The process of mitochondrial fission can be further enhanced through mild cyclic strain, which is a response to support energy production. Furthermore, morphological changes and mitochondrial elongation alter respiration rates and enhance oxidative phosphorylation through PI3K‐Akt signaling when cells are cultured on stiffer substrates.^[^ ^144^, ^145^ ^]^ Mitochondria can also be transported along microtubules to high‐tension regions, such as the leading edges during focal adhesion formation. This process was linked to kinesin‐and dynein‐based mechanisms. Alteration of mitochondria can also modulate ROS generation, calcium buffering, and energy supply to cells, influencing cell adhesion, proliferation, and survival.

**Figure 5:**
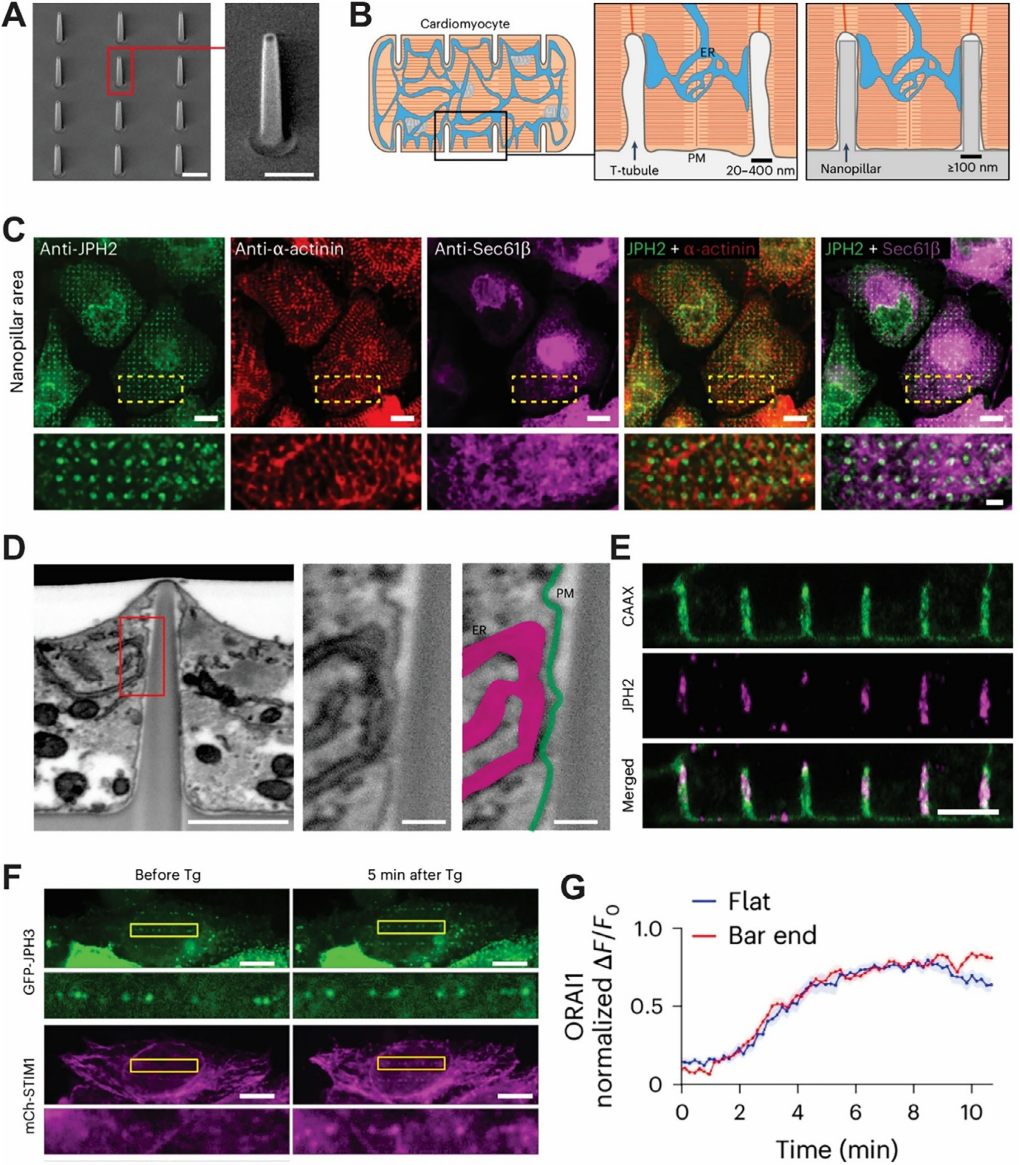
A) Scanning electron microscopy (SEM) images showing the structure of nanopillars. The left image includes a scale bar of 1 µm, and the right shows finer detail with a 500 nm scale bar. B) Illustrations depicting: (left) the T‐tubule architecture in a cardiomyocyte, (middle) endoplasmic reticulum–plasma membrane (ER–PM) junctions at T‐tubules, and (right) membrane invaginations triggered by nanopillar interaction. C) Immunofluorescent staining of JPH2 (green) and α‐actinin (red) in iPSC‐derived cardiomyocytes expressing GFP‐Sec61β (magenta), cultured on nanopillar arrays (d). Enlarged insets from boxed regions are shown below. Scale bars: 10 µm (top images), 2.5 µm (bottom photos). D) Left: Inverted FIB‐SEM image of an iPSC‐CM–nanopillar interface. Scale bar: 1 µm. Middle: Close‐up of the red‐boxed region. Scale bar: 100 nm. Right: Same close‐up with the plasma membrane (green) and ER (magenta) shown in pseudo‐color. E) The x‐z view focused on the middle height of nanopillars using an expansion microscope. Scale bar, 10 µm. F) U2OS cell line after co‐transfection with mCherry‐STIM1 and GFP‐JPH3 before or 5–10 min after treating with 10 µm thapsigargin (Tg). Tg leads to mCherry‐STIM1 accumulation at the end of the nanobars. Zoomed‐in views of the regions are highlighted in yellow boxes, and scale bars are 10 µm (zoomed‐out view) and 5 µm (zoomed‐in view). (G) Time‐dependent increases (normalized as ΔF/F0) of ORAI1 cluster fluorescence intensities at the end of the bar versus flat area in GFP‐ORAI1 transfected cells after Tg treatment (regions of interest: 0.85 × 0.85 µm^2^). *n* = 21 regions selected from seven cell images. Reproduced with permission.^[^ ^148^ ^]^ Copyright 2024, Nature Publishing Group.

In parallel, the endoplasmic reticulum (ER) responds to mechanical signals via ER membrane deformation, calcium flux, and activation of proteostasis pathways. Actomyosin contraction and cell shape changes impose membrane tension on the ER, which triggers calcium release through mechanosensitive channels like IP3R and RyR at the timescale of 10–60 s.^[^ ^146^ ^]^ These local calcium spikes regulate actomyosin contractility and focal adhesion dynamics, coupling cytoskeletal behavior to intracellular signaling. Under sustained mechanical stress (e.g., stiff ECM or confined migration), the ER undergoes structural remodeling, shifting from sheet‐like to tubular architecture, within 30–60 min.^[^ ^147^ ^]^ Concurrently, the unfolded protein response (UPR) is activated over 1–3 h through PERK, ATF6, and IRE1 pathways, modulating transcription of chaperones and protein‐folding enzymes. The ER also forms mechanosensitive contact sites with mitochondria (MAMs), facilitating calcium and lipid exchange in response to force. These interactions coordinate metabolic adaptation and survival responses during mechanically demanding processes like migration, fibrosis, or epithelial morphogenesis. In another study by Cui et al. (**Figure** **5**), they revealed how plasma membrane (PM) curvature influences the formation of endoplasmic reticulum (ER)–PM contact sites in cardiomyocytes.^[^ ^148^ ^]^ They observed that ER–PM contacts preferentially form in PM regions with high curvature, particularly within the transverse tubule system of cardiomyocytes. The junctophilin family of ER–PM tethering proteins, specifically expressed in excitable cells, plays a central role in this process. The study identified that both the low‐complexity region (LCR) and membrane occupation and recognition nexus (MORN) motifs of junctophilins are necessary for targeting PM curvatures. Moreover, the researchers discovered that Eps15 homology domain‐containing proteins, which are curvature‐sensing, interact with the MORN_LCR motifs of junctophilins, facilitating their preferential localization to curved PM regions. This work highlights the significance of PM curvature in the spatial regulation of ER–PM contacts and provides insights into the molecular mechanisms underlying this process. Understanding how PM curvature influences ER–PM contact formation could have implications for diseases associated with disrupted ER–PM interactions, such as cardiovascular and neurodegenerative disorders.

Lipid droplets and associated metabolic machinery act as mechanosensitive hubs that modulate membrane synthesis and energy storage. Substrate stiffness and cytoskeletal contractility activate RhoA–ROCK signaling, triggering Lipin‐1 nuclear translocation and enhancing SREBP processing in the Golgi over 2–4 h. These transcription factors upregulate enzymes involved in fatty acid synthesis and membrane lipid remodeling, preparing the cell for membrane expansion or organelle growth. Lipid droplets (LDs), synthesized at the ER, dynamically increase in size and number within 1–3 h following mechanical stress, particularly in compressed or high‐tension environments. LDs buffer excess lipid load and serve as metabolic reservoirs that support membrane tension regulation and autophagy during force‐induced remodeling. Mechanical stretch alters membrane curvature and local lipid composition, affecting signaling platforms and anchorage of cytoskeletal proteins, contributing to feedback loops in mechanotransduction.

Lastly, the Golgi apparatus functions as a central hub for protein trafficking, membrane lipid processing, and glycosylation. Although not directly involved in mechanotransduction, many functions of the Golgi apparatus are dynamically regulated by mechanical signals.^[^ ^149^ ^]^ For example, Golgi positioning is highly responsive to cytoskeletal tension and mechanical stimuli, which can induce Golgi fragmentation or reorientation (typically toward the leading edge in migrating cells) within 30–90 min, driven by microtubule and actin cytoskeletal remodeling. Mechanosensitive trafficking through the Golgi could be modulated via ARF1, Rab GTPases, and the COPI/COPII machinery, which respond to changes in tension and curvature of Golgi cisternae.^[^ ^150^ ^]^ Furthermore, Golgi‐mediated post‐translational modifications such as glycosylation of integrins, cadherins, and ECM components are dynamically regulated by matrix stiffness over several hours, altering cell adhesion and signaling. The Golgi also interfaces with the ER in stress conditions, helping manage load through ER–Golgi intermediate compartments (ERGIC) and secretory flux.^[^ ^151^ ^]^ Nevertheless, further research is needed to elucidate the precise mechanisms directly linking the Golgi apparatus to mechanotransduction.

In living systems, cells show diverse mechanobiological dynamics in response to external forces and a complex array of biological stimuli (**Table** **1**). These stimuli encompass chemical, mechanical, thermal, electrical, and enzymatic cues critical for maintaining physiological balance and driving adaptive responses.^[^ ^94^, ^152^, ^153^ ^]^ Understanding how cells interpret and respond to these signals is at the heart of cell biology, regenerative medicine, and mechanobiology. From the perspective of mechanobiology, these stimuli do not act in isolation. Instead, they are integrated through complex signaling networks that sense, process, and respond to the combined chemical and physical landscape of the cell's environment. The extracellular matrix (ECM) acts as a crucial mediator of this integration, offering both structural support and biochemical cues. Cells dynamically sense and respond to the stiffness, composition, and topography of their microenvironment via focal adhesions and the actomyosin cytoskeleton. These mechanical interactions influence signal transduction pathways like RhoA/ROCK, YAP/TAZ, and MAPK, which regulate gene expression and cellular behavior. As such, cells within physiological environments are continually exposed to a diverse array of biological stimuli, including oxidative stress, inflammation, hypoxia, mechanical forces, temperature fluctuations, osmotic pressure, nutrient availability, bioelectrical signals, and enzymatic activities. These stimuli are intricately interconnected, and their integration is critical for maintaining homeostasis, guiding development, and responding to injury. Mechanobiology provides a powerful framework for understanding how mechanical and biochemical signals are sensed, integrated, and processed within cells and tissues. By elucidating the fundamental principles that govern cellular and tissue function, this framework also offers critical insights into processes such as development, disease progression, and tissue regeneration, thereby guiding the design of innovative biomaterials and therapeutic strategies.

**Table 1:** A summary of mechanobiological dynamics commonly seen in cells.

Process	Timescale	Process	Timescale
Fibrillogenesis (e.g., collagen fiber formation)	Seconds–minutes	DNA base‐pair vibrations	Femtoseconds–picoseconds (fs–ps)
Fibronectin unfolding under tension	Milliseconds–seconds	Nucleosome breathing (DNA unwrapping)	Milliseconds–seconds (ms–s)
ECM stiffening via cross‐linking (e.g., LOX)	Minutes–hours	Nucleosome sliding/remodeling	Seconds–minutes
Elastic deformation of ECM fibers	Milliseconds–seconds	Chromatin fiber folding/unfolding	Milliseconds–minutes
Matrix metalloproteinase (MMP) activation	Seconds–minutes	Chromatin domain movement (e.g. TADs)	Seconds–minutes
Collagen or elastin degradation	Minutes–hours	Epigenetic modification (e.g., methylation)	Minutes–hours
ECM turnover (in tissues)	Hours–days	Process	Timescale
Elastic force propagation	Milliseconds	Protein diffusion (e.g., transcription factors)	Milliseconds–seconds
Tension‐induced conformational changes	Milliseconds–seconds	Target search by TFs (facilitated diffusion)	Seconds
Cell‐generated force transmission	Milliseconds–seconds	Binding/unbinding to DNA	Milliseconds–seconds
ECM strain stiffening	Seconds–minutes	Phase separation of nuclear bodies (e.g., nucleoli, speckles)	Seconds–minutes
Lipid tail motions	Picoseconds–ns	Nuclear pore complex (NPC) transport	Milliseconds–seconds
Lipid lateral diffusion	Microseconds–ms	Nuclear envelope deformation (under force)	Milliseconds–seconds
Lipid flip‐flop	Minutes–hours	NE breakdown/reassembly (mitosis)	Minutes
Protein side chain motions	Picoseconds–ns	DNA base‐pair vibrations	Femtoseconds–picoseconds (fs–ps)
Protein conformational changes	Microseconds–s	Protein translocation into ER lumen	Seconds–minutes
Protein diffusion	Milliseconds–s	Protein folding (chaperone‐assisted)	Seconds–minutes
Lipid raft formation	Milliseconds–s	ER membrane protein diffusion	Milliseconds–seconds
Vesicle fusion/budding	Seconds–minutes	ER Ca^2^⁺ release (e.g., via IP3R)	Milliseconds–seconds
Elastic deformation of lipid bilayer	Femtoseconds–nanoseconds (fs–ns)	ER remodeling/movement	Seconds–minutes
Propagation of tension across membrane	Microseconds–milliseconds (µs–ms)	Vesicle budding and fusion (cisternal traffic)	Seconds–minutes
Protein conformational response to force (e.g., mechanosensitive channels)	Microseconds–milliseconds	Glycosylation and protein processing	Minutes
Transmembrane signal propagation (e.g., via integrins or cadherins)	Milliseconds–seconds	Golgi stack reorganization	Minutes–hours
Monomer fluctuations (G‐actin)	Picoseconds–nanoseconds (ps–ns)	Cargo transport through Golgi	Minutes
Actin filament bending fluctuations	Nanoseconds–microseconds (ns–µs)	ATP synthesis (oxidative phosphorylation)	Milliseconds
Polymerization/depolymerization	Seconds–minutes	Mitochondrial fission/fusion	Seconds–minutes
Treadmilling (net flux of subunits)	Tens of seconds–minutes	Protein import into mitochondria	Seconds–minutes
Severing by proteins (e.g., cofilin)	Seconds	ROS production and scavenging	Milliseconds–seconds
Cross‐linking and bundling	Milliseconds–seconds	Membrane potential fluctuation	Milliseconds–seconds
Force generation (e.g., pushing membrane)	Milliseconds–seconds	Fusion with autophagosomes/endosomes	Seconds–minutes
Monomer fluctuations (G‐actin)	Picoseconds–nanoseconds (ps–ns)	Enzymatic degradation of cargo	Minutes–hours
Actin filament bending fluctuations	Nanoseconds–microseconds (ns–µs)	Lysosome movement	Seconds–minutes
Polymerization/depolymerization	Seconds–minutes	pH regulation (via V‐ATPase)	Seconds
Tubulin monomer dynamics	ps–ns	Peroxisomal protein import	Seconds–minutes
Dynamic instability (growth/shrinkage)	Seconds–minutes	β‐oxidation of fatty acids	Seconds–minutes
Catastrophe (sudden disassembly)	Milliseconds–seconds	ROS detoxification (e.g., catalase)	Milliseconds–seconds
Rescue (re‐growth)	Milliseconds–seconds	Peroxisome proliferation/fission	Minutes–hours
Motor protein movement (e.g., kinesin)	Milliseconds	Endocytosis and vesicle formation	Milliseconds–seconds
Nucleation and anchoring	Minutes	Early‐to‐late endosome maturation	Minutes
Subunit fluctuations	ps–ns	Cargo sorting and recycling	Seconds–minutes
Polymerization	Minutes–hours	Endosome‐lysosome fusion	Seconds–minutes
Reorganization under stress	Seconds–minutes	Adhesion initiation	ms–s
Assembly/disassembly (slow)	Minutes–hours	Adhesion maturation	min–hours
Cross‐linking and mechanical resistance	Seconds–minutes	Cell spreading	min–hours
Myosin (actin motor)	Power stroke, ATP cycle	Cell cycle (proliferation)	12–30 h
Kinesin/Dynein (microtubule motors)	Stepping, cargo transport	Mitosis	≈30–90 min
Cross‐linkers (e.g., filamin, spectrin)	Binding/unbinding dynamics	Differentiation	Hours–days (sometimes weeks)
		Apoptosis	Minutes–hours
		Migration	Minutes–hours (up to days)
		Senescence onset	Hours–days
		Polarization	Minutes–hours

## 4 Stimuli‐Responsive Biomaterials for Dynamic Mechanomodulation

**Figure 6:**
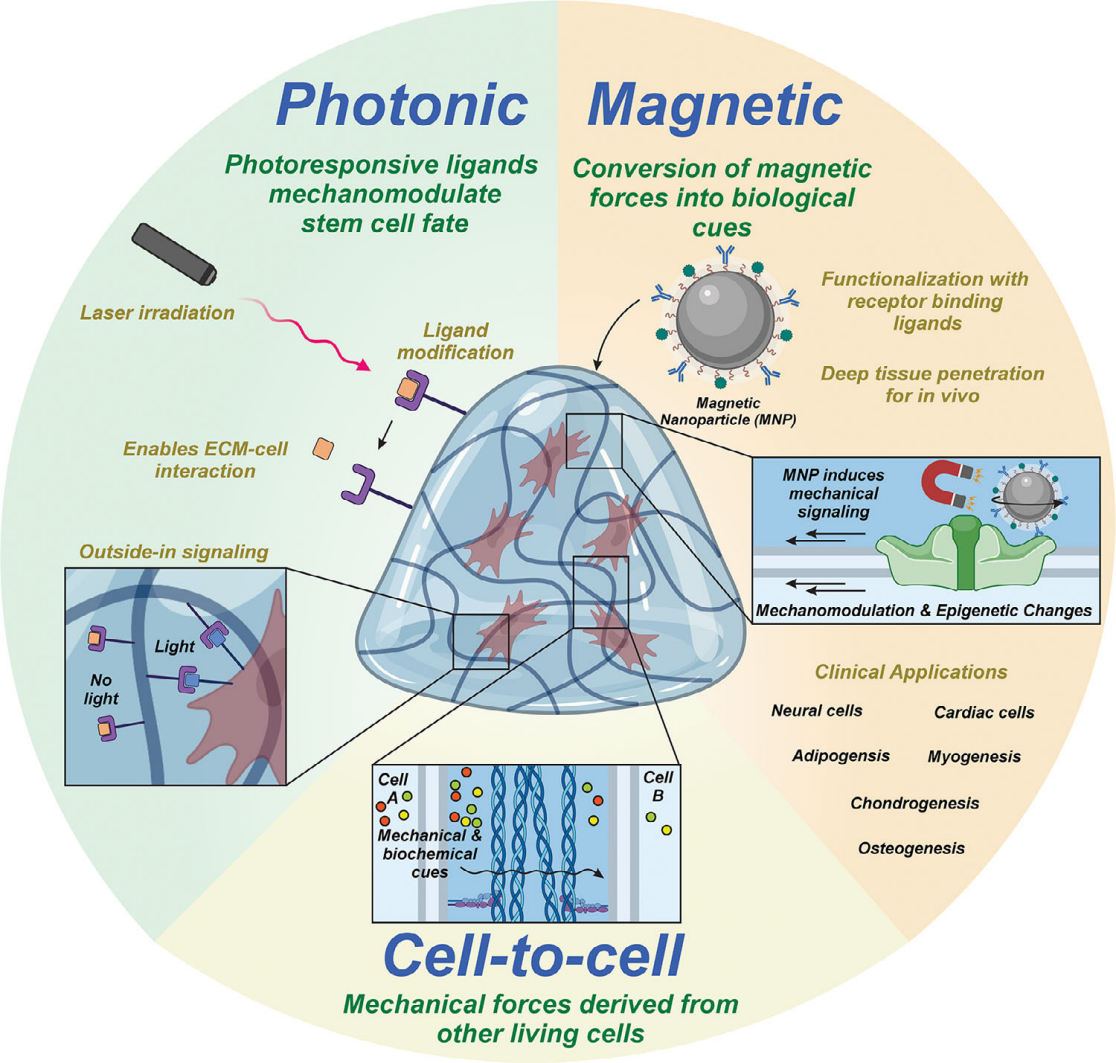
A summary of dynamic mechanomodulation of cell fates by stimuli‐responsive biomaterials. This scheme demonstrates how programmable biomaterials responsive to diverse physical stimuli—including mechanical forces, light, magnetic fields, and ultrasound—enable dynamic control over cellular behaviors and fate specification. External actuation triggers reversible material property changes in stiffness, nanoscale topography, and biochemical signaling presentation. Cells decode these dynamic mechanical cues through specialized sensory apparatus, including integrin‐based adhesions, mechanosensitive channels, and cytoskeletal networks, converting physical inputs into downstream signaling cascades that direct cellular responses. These mechanical cues activate intracellular signaling pathways, leading to alterations in gene expression and subsequent cell responses, such as proliferation, differentiation, and migration. The scheme highlights the versatility of stimuli‐responsive biomaterials in creating dynamic microenvironments that can be tailored to guide specific cellular outcomes in tissue engineering and regenerative medicine applications.

From the previous section, we have concluded that there are diverse types of mechanobiological dynamics in varying cell organelles. These mechanobiological dynamics are delicately regulated by intrinsic stimuli within the cells and the microenvironment. In addition to intrinsic stimuli, stimuli‐responsive biomaterials can also deliver dynamic mechanobiological signals to regulate cellular behaviors.^[^ ^154^, ^155^ ^]^ Understanding the effects of these highly dynamic interactions on cellular fates has received tremendous interest from physicists, biologists, and bioengineers in the past decade. These efforts have led to a better understanding of organogenesis, cancer metastasis, and stem cell fate control. Nevertheless, how to leverage these breakthroughs in the 3D cell‐tissue interactions over varying periods of time to improve stem cell fate control in vitro and in vivo is still an ongoing challenge in tissue engineering and mechanobiology. Integrating advancements in dynamic material design and innovation to recapitulate the unique dynamic ligand‐receptor interactions in a spatiotemporally controlled manner is thus at the frontier of stem cell‐based regenerative medicine (**Figure** **6**).^[^ ^137^, ^156^ ^]^

To recapitulate the dynamic modulation of cell‐tissue interactions in vitro, different signals have been used for achieving temporal control over ECM or cell‐ECM interactions. These signals have been generated by all major types of energy sources, including photonic, electrical, magnetic, thermal, and acoustic sources. However, at the cell membrane, these varying types of energies would eventually be converted into mechanical forces that either directly trigger biomolecular responses intracellularly or remodel the cell membrane and cytoskeletal structures to initiate downstream mechanotransduction pathways. In this section, we will summarize how various energy sources mechanomodulate stem cell fates and examine the underlying mechanisms involved in stem cell fate regulation.

### 4.1 Photoresponsive Biomaterials for Dynamic Mechanomodulation of Cell Fates

Light has become one of the most widely utilized energy sources, offering superior spatial resolution for controlling cell fate both in vitro and in vivo, thus demonstrating significant potential for mechanobiology studies and regenerative medicine. In fact, photochemistry naturally exists in nature, such as the photoreceptor cells in the formation of human eyes. Recent advancements in optogenetics further substantiate light as an external source of manipulating cells, including controlling stem cell differentiation.^[^ ^157^ ^]^ As such, there has been enormous interest in attempting to control biological responses and cell fate in 4D by using various types of light‐driven reactions. Moreover, as stem cell fates can be regulated by multiple mechanotransduction pathways, the ability to use different wavelengths to orthogonally manipulate stem cell pathways or surface receptors is particularly attractive. The key challenge, however, is on how to accurately tether photoresponsive ligands to cell membranes or surrounding ECM, and how to leverage these photoresponsive ligands to mechanomodulate stem cells in situ and in a dynamic manner.

**Figure 7:**
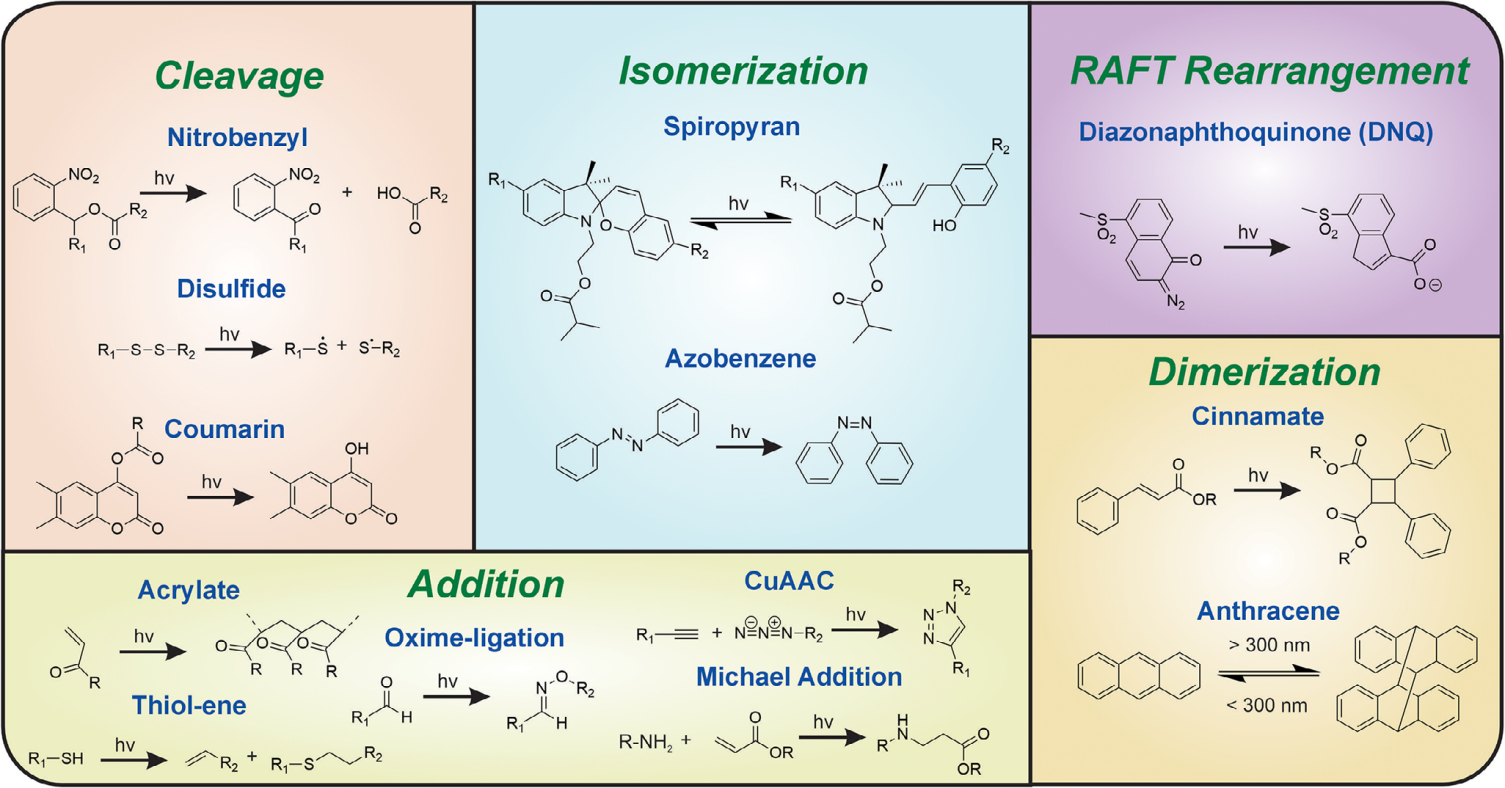
A summary of chemical reactions used in the synthesis of photoresponsive molecules.

Thanks to the efforts made by chemists, there has been a well‐established library of synthetic materials controllable by light sources in a spatiotemporally and chemically predictable, and tunable manner. These materials rely on light triggerable cleavage^[^ ^158^ ^]^ (e.g., nitrobenzyl,^[^ ^159^ ^]^ disulfide,^[^ ^160^ ^]^ and coumarin^[^ ^161^ ^]^)‐, addition (e.g., acrylate,^[^ ^162^ ^]^ thiol‐ene,^[^ ^163^ ^]^ oxime ligation,^[^ ^164^ ^]^ CuAAC,^[^ ^165^ ^]^ Michael addition,^[^ ^166^ ^]^ and factor XIII (FXIII) catalyzed conjugation^[^ ^167^ ^]^)‐, isomerization (e.g., spiropyran^[^ ^168^ ^]^ and azobenzene^[^ ^169^ ^]^)‐, diazonaphthoquinone (DNQ)/reversible addition‐fragmentation chain transfer (RAFT) rearrangement‐,^[^ ^170^ ^]^ and dimerization (e.g., cinnamate^[^ ^171^ ^]^ and anthracene^[^ ^172^ ^]^)‐based photochemistry (**Figure** **7**). Once properly tethered to ligands that bind to mechanosensing receptors of stem cells, these ligands can activate intracellular mechanotransduction pathways for stem cell fate control. In general, based on the mechanism of action (MOA), light‐triggered mechanomodulation can include the biochemical and biophysical pathways, which alter integrin‐bound ligand density and stiffness of ECM, respectively.

Modulation of ligand densities (e.g., arginylglycylaspartic acid, or RGD) in hydrogels has been an effective approach to regulating stem cell proliferation and differentiation. Photo‐initiated conjugation of integrin‐binding ligands has been widely adapted in hydrogel synthesis. In the current review, the 4D mechanomodulation system is defined as having the ability to introduce and detach these ligands in situ reversibly. Specifically, PEG‐based hydrogels synthesized from thiol‐ene cross‐linking often contain free alkene ligands for adding thiol groups.^[^ ^173^ ^]^ Therefore, after stem cell encapsulation by PEG hydrogels, RGD densities can be increased in situ for the dynamic modulation of stem cell differentiation.^[^ ^174^ ^]^ If PEG hydrogel is bridged by nitrobenzyl groups, the exposure of the hydrogel to ultraviolet (UV) light can then controllably cleave the nitrobenzyl groups, thus detaching RGD domains that can also dynamically remodel stem cells.^[^ ^175^ ^]^ Furthermore, besides these two modes (addition or attachment of RGD), RAFT reactions have enabled the iterative conjugation of thiol‐containing RGD. However, the dependence on free radicals restricts the spatial resolution when controlling stem cell fate. Bioorthogonal oxime ligation, which allows for the conjugation and cleavage of nitrobenzyl photocleavable group‐functionalized biomolecules, can have better spatial control in this regard. For instance, the spatially controlled grafting and cleavage of vitronectin reversibly regulate the osteogenesis of MSCs encapsulated within the hydrogel.^[^ ^176^ ^]^ Nevertheless, the reversibility of this cross‐linking system seems to be limited within a few cycles, necessitating future development of more advanced chemistry and a new generation of reversible hydrogel systems.

Another strategy to develop light‐mediated dynamic mechanomodulation is through the reversible modulation of hydrogel stiffness. Through decoration with functional groups that can dimerize and dissociate in a reversible manner, hydrogels with reversible mechanical properties have been prepared and applied for the modulation of stem cell differentiation. Cinnamylidene acetyl groups grafted to PEG hydrogels, for example, dimerize under exposure to a 375 nm laser and dissociate under a 254 nm laser, leading to reversible control over hydrogel stiffness.^[^ ^177^ ^]^ Coumarin and anthracene ligands also have similar dual‐wavelength responsive properties and have a potential for dynamic mechanomodulation of hydrogels,^[^ ^178^, ^179^ ^]^ although their reliance on cytotoxic UV wavelengths (e.g., sub‐300 nm wavelength) limits their broad applications. Azobenzene‐based systems require less toxic laser exposure, but the stiffness change is only moderate (≈200 Pa).^[^ ^180^ ^]^

In general, light‐based 4D mechanomodulation of stem cells has demonstrated excellent potential in terms of high spatial resolution and adaptability for 3D hydrogel systems. However, the limited tissue penetration of UV light would challenge their practical applications in vivo. Incorporating upconversion nanoparticles (UCNP), or developing near‐infrared (NIR)‐responsive reversible ligands, may be possible routes to circumvent this limitation. The range of mechanical properties available in photoresponsive hydrogels, as well as the reversibility of these hydrogels, remains largely unsatisfactory, representing additional challenges, but also opportunities for future research in this exciting new direction.

### 4.2 Magnetic Biomaterials for Dynamic Mechanomodulation of Cell Fates

Magnetic nanomaterials, including 0, 1, 2, and 3D nanostructures, have broad applications in biomedical engineering, including MRI imaging, biomolecule isolation, cell sorting, hyperthermia‐based cancer therapy, and also mechanomodulation of stem cells. Compared to light‐based actuation systems, magnetic forces have much deeper tissue penetration, which is critical for potential in vivo applications. Also, magnetic nanoparticles can be surface‐engineered with receptor‐binding ligands, allowing target‐specific modulation of stem cells. In nature, magnetic field‐based modulation of cells that guide the migration of animals, also known as magnetoreception,^[^ ^181^ ^]^ further highlights the potential of a remote and dynamic mechanomodulation control of cells in vivo through the conversion of magnetic forces into biological cues. However, due to the minimal innate magnetic sensitivity of cells and cell membrane receptors, magnetic force‐based modulation of stem cells has not been well developed.

**Figure 8:**
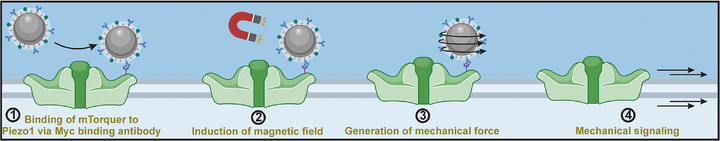
Schematic diagram showing the magnetic field‐induced mechanomodulation of stem cells.

Addressing this challenge, a promising approach is to combine cell‐targeted magnetic nanoparticles to enhance the magnetic sensitivity of cells (**Figure** **8**). In the past three decades, chemists have made significant contributions to synthesizing magnetic nanomaterials with varying sizes, compositions, and shapes. Robust methods to functionalize these magnetic nanomaterials with cell‐targeting ligands have also been developed to realize target‐specific cancer therapy, drug and gene delivery, and magnetothermal modulation of neural cells.^[^ ^182^, ^183^, ^184^ ^]^ There have been excellent reviews on this topic, and they have paved the road for the further development of remote magnetic mechanomodulation of cells in vivo. The realization of magnetic nanomaterial‐based 4D mechanomodulation of stem cell fates requires joint efforts by chemists, material scientists, and bioengineers. For example, further improvement in terms of the design and synthesis of magnetic nanomaterials is necessary for the translation into large animal models or the treatment of human diseases.

Cheon et al. developed a magnetic nanomaterial‐based 4D dynamic mechanomodulation system for in vivo activation of neural cells.^[^ ^185^ ^]^ To endow cells with mechanical sensitivity, target cells were genetically encoded with Piezo1 channels that would release calcium ions in response to mechanical stimulation. To improve the sensitivity of cells to magnetic fields, cells with Piezo1 channels were also transduced to express Myc tags, which can bind to magnetic nanoparticles functionalized with Myc‐binding antibodies. This also facilitates target‐specific mechanomodulation by magnetic fields. In parallel, the design of magnetic nanomaterials was carefully optimized to maximize their mechanical responsiveness to external magnetic fields. Last, for in vivo applications, it is equally important to precisely tune the magnetic field, requiring advancements in magnetic field generators.

Specifically, they developed a magnetic torquer nanomaterial (mTorquer) that is composed of small (≈25 nm) octahedral nanoparticles assembled on the surface of a spherical microparticle (≈500 nm). Through this design, the octahedral magnetic nanoparticle with 111 face exposed promotes the anisotropy of the hybrid particle and significantly enhances the overall ferromagnetism of the particle, with over 470 times larger saturation magnetization while keeping a similar coercivity. Also, because of the weak ferromagnetism, the particle remains stable in solution for over a month. Within a circular magnet array (CMA), mTorquer can generate 2 – 10 pN forces at a 20 – 50 mT magnetic field, which suffices to induce activation of Piezo1 mechanosensitive channels. This corresponds to an interaction energy of 1.38 × 10^−17^ J – nearly four orders of magnitude higher than the thermal energy, thus differentiating itself from previous studies using genetically coded magnetic nanoparticles with much weaker interaction. However, it would still be a challenge to create a large‐scale magnetic field that is applicable to in vivo mechanomodulation of mice. Therefore, increasing the number of magnets within the array to 10 magnets eventually allows for a uniform 20 mT magnetic field up to 70 cm container, which is comparable to a human MRI instrument. This large‐scale uniform field with magnetic gradients less than 10 T m^−1^ would avoid unnecessary interference with animal behaviors. Also, within the CMA field, the random orientation of the octahedral magnetic nanoparticles within the spherical particle results in a minimum pulling force on the cells. Still, it maintains susceptibility to the torque forces during the rotation of the field.^[^ ^185^ ^]^

Next, the CMA‐based 4D mechanomodulation was tested in a neuron‐based in vitro system and then in vivo. Primary cortical neurons were transduced with an adenoviral vector to achieve robust expression of Piezo1 channels at a density of ≈4 channels per µm^2^. The magnetic nanotransducer mTorquer was then conjugated to an anti‐Myc antibody and specifically immobilized on the neuronal surface via immunoaffinity binding to Myc‐tagged Piezo1 channels. Dynamic mechanomodulation of cells can then be achieved by setting the frequency of the magnetic field, initially at 0.5 Hz. Compared to cells only treated by CMA or mTorquer, only mTorquer combined with CMA, triggered neuron activation, as evidenced by the expression of c‐Fos mRNA within the nucleus. In addition, the treatment of ruthenium red, which is the inhibitor of Piezo1, or the use of wheat germ agglutinin‐bound mTorquer, which binds to the cell membrane non‐specifically, also abolished the c‐Fos expression triggered by CMA. A similar trend was observed in the calcium influx experiment. Remarkably, this whole rotation and mechanoactivation process does not trigger any magnetothermal effects or a significant decrease in cell viability. For the 4D in vivo mechanomodulation of mice, cells in the motor cortex were transduced with Piezo1‐expressing adeno‐associated viruses (AAVs). Then, 4 weeks later, mTorquer was treated to animals through intracranial injection. Through further dynamic stimulation by CMA at a frequency of 0.5 Hz, c‐Fos expression within the mouse brain was significantly upregulated, accompanied by increased movement within the magnetic field, as compared to non‐stimulated or those without treatment of m‐Torquer.^[^ ^185^ ^]^

Beyond 4D mechanomodulation of neural cells, magnetic nanoparticles have been applied for regulating MSCs and ESCs adipogenesis,^[^ ^186^ ^]^ myogenesis,^[^ ^187^ ^]^ chondrogenesis,^[^ ^188^ ^]^ and osteogenesis,^[^ ^189^ ^]^ as well as cardiac cell maturation,^[^ ^190^ ^]^ through mechanisms such as integrin clustering, cytoskeletal modulation LINC, and transcriptional alteration. Magnetic forces can be converted into biologically active forces through stretching, twisting, or pulling. Nonetheless, most of the previous systems have not yet provided an in‐depth understanding of the link between the dynamics of magnetic force application and stem cell differentiation, including the mechanomodulation mechanisms behind it. Another promising direction is large‐scale magnetic field‐based mechanomodulation of stem cells at piconewton precision. Realizing this goal would significantly facilitate the translation of magnetic force‐based mechanotransduction studies and stem cell fate determination, but also require major technical breakthroughs.

### 4.3 Mechanical Forces Generated by Living Cells for the Mechanomodulation of Cell Fates

Another excellent example of the 4D mechanomodulation of stem cells is through forces generated by surrounding cells, which are often dynamic with varying frequencies. Mechanical cues, together with soluble factors (e.g., morphogens), are essential for embryogenesis. For example, poking frog eggs directly leads to fertilization. Given the high cell densities within the embryo and human tissues, intercellular forces modulate the stem cell fates and the embryo tissue development, from the generation of initial tissue shapes, patterning of biomechanical microenvironment, breaking of tissue symmetry, to the tissue boundary formation.^[^ ^191^, ^192^, ^193^ ^]^ Forces generated by surrounding cells act on stem cells through motor proteins (actomyosin or filopodia) that are linked to the cytoskeleton, which can directly modulate the motility of stem cells, such as oocytes’ migration during the wound healing process of Xenopus laevis.^[^ ^194^ ^]^ Bottle cells restricted by the apical plane induce tissue involution surrounding the blastopore.^[^ ^195^ ^]^ These cell‐generated forces then integrate into the tissue scale, leading to the coordination of cytoskeletons and convergent extension. This mechanical pattern can be further reinforced by recruiting tension‐sensitive proteins such as myosin. In the event of cavity formation, internal hydrostatic pressure will then coordinate with the cellular forces to form the blastocoel through ion pump‐related activities. Hydraulic fracturing could also occur due to the build‐up of internal hydrostatic pressure. With the influx of water and breakup of intercellular forces, the stretching of the external trophectoderm can increase cortical tension until reaching a force balance. Mechanical feedback between the pressure inside the cavity and the maximally sustainable pressure from the trophectoderm determines the final size of embryos during development.^[^ ^196^ ^]^

4D dynamic mechanical forces generated by surrounding cells can also regulate stem cell differentiation during vertebrate development by generating morphogen gradients. Morphogen gradient is the most studied signaling mechanism for defining tissue patterns containing multiple cell types during embryogenesis. For example, villi formation within the mouse gut is mediated through an sonny hedgehog (SHH) gradient, with tip cells generating more SHH.^[^ ^197^ ^]^ Other crucial morphogen gradients discovered so far include WNTs, FGFs, TGFβ superfamily of ligands (e.g., ACTIVIDIN/NODAL and BMP). Mechanical stimuli have been shown to modulate these crucial morphogen secretions, and thus tremendous efforts have been made to study the role of the mechanical cue gradient generated by dynamic cell forces on tissue patterning.^[^ ^198^ ^]^ Indeed, YAP/TAZ has been identified as the key mediator of the differential expression of morphogens in response to different forces generated by surrounding cells. This mechanism has been associated with the cortical rotation process during dorsoventral axis specification and left‐right patterning during vertebrate development.^[^ ^199^ ^]^ Spatial patterning of embryonic and extra‐embryonic tissues is also mediated by differential YAP/TAZ activation in the apical domain, which originates from blastocyst compaction and leads to the activation of transcription factors CDX2 and SOX2 in the trophectoderm and inner cell mass, respectively.^[^ ^200^ ^]^

Overall, the cell force‐mediated mechanomodulation of stem cells during embryogenesis is still under rapid development (**Table** **2**). Active efforts are made to develop in vitro tissue models to study morphogenesis determined by mechanical cues. However, leveraging these highly dynamic cell‐generated forces on precise tissue engineering remains a critical challenge. Using synthetic cells that can recapitulate key mechanical modulation effects from living cells while preserving better control over force generation could be a promising direction. Furthermore, it is crucial to elucidate the dynamic roles of 4D mechanical cues during embryogenesis to advance our understanding of human embryonic development and related genetic disorders of tissue morphogenesis. Leveraging recent advances in in vitro 3D morphogenesis models offers a powerful approach for these investigations.

**Table 2:** Summary of stimuli, biomaterials, and response outcomes in mechanbiology studies.

Cell	Vitro/Vivo	Material	Stimuli	MOA	Cell fate	References
Conjunctive MSCs	In vitro/human	PCL/PPY nanofibers	Electrical	Modulation of channel ion channel distributions	Differentiation	^[^ ^214^ ^]^Abdar, Z.E.et al. *International Journal of Artificial Organs* **45**, 695–703 (2022).
BMSC	In vitro/ human	Collagen graphene cryogel	Electrical	Modulation of channel ion channel distributions	Differentiation	^[^ ^215^ ^]^Agarwal, G., Kumar, N. & Srivastava, A. *Materials Science & Engineering C‐Materials for Biological Applications* **118**(2021).
PC‐12	In vitro/ rat	rGO	Electrical	NA	Differentiation	^[^ ^216^ ^]^Aznar Cervantes, S.*, et al. Materials Science and Engineering C‐Materials for Biological Applications* **79**, 315–325 (2017).
MSCs	In vitro/rat	PVA/PEDOT scaffolds	Electrical	Protein adsorption	Differentiation	^[^ ^217^ ^]^Babaie, A.*, et al. European Polymer Journal* **140**(2020).
SH‐SY5Y	In vitro/human	PEDOT:PSS	Electrical	Modulation of channel ion channel distributions	Differentiation	^[^ ^218^ ^]^Bonisoli, A., Marino, A., Ciofani, G. & Greco, F. *Macromolecular Bioscience* **17**(2017).
ADMSCs	In vitro/porcine	Polyaniline nanofiber – chitosan anocomposite	Electrical	Integrin signaling and ion channel modulation	Differentiation	^[^ ^219^ ^]^Borah, R., Das, J.M. & Upadhyay, J. *Acs Applied Bio Materials* **5**, 3193–3211 (2022).
PC12/RSC96/ BMMSC	In vitro & in vivo	Sodium alginate and carboxymethyl chitosan hydrogel	Electrical	Promotes axonal growth	Differentiation	^[^ ^220^ ^]^Bu, Y.*, et al*. *Rsc Advances* **8**, 10806–10817 (2018).
NPC	In vitro mouse	Microfluidic device	Magnetic	NA	Differentiation	^[^ ^221^ ^]^1). Chang, H.F., et al. *Jove‐Journal of Visualized Experiments* (2021). ^[^ ^222^ ^]^2). Chang, H.F., et al. *Plos One* **11**(2016).
BMSCs	In vitro and in vivo rabbit	PLA nanofiber	Electrical	Enhanced osteoblasts‐scaffold affinity	Differentiation	^[^ ^223^ ^]^Chen, J., et al. *Journal of Colloid and Interface Science* **514**, 517–527 (2018).
rBMSCs	In vivo rat	PLA nanofibers w/piezo electric niobate nanowire	Ultrasound	Enhanced neurotrophic factor production	Differentiation	^[^ ^224^ ^]^Chen, P.*, et al. Acs Nano* **16**, 16513–16528 (2022).
BM‐MSCs	In vitro human	PEG‐phospholipid @magnetite nanoparticles	Electromagnetic	Phosphorylation of CREB	Differentiation	^[^ ^225^ ^]^Choi, Y.K.*, et al. Applied Biochemistry and Biotechnology* **174**, 1233–1245 (2014).
MSCs	In vitro/ rat	Graphene	Electrical	Modulation of channel ion channel distribution & Focal adhesion kinase signaling (FAK)	Differentiation	^[^ ^226^ ^]^Das, S.R.*, et al. Advanced Healthcare Materials* **6**(2017).
NSCs	In vitro/ mice	Graphene oxide (GO)	Electrical	Cell‐biomaterial interaction	Differentiation	^[^ ^227^ ^]^Fu, C.*, et al. Artificial Cells Nanomedicine and Biotechnology* **47**, 1867–1876 (2019).
iPSC‐NSCs	In vitro/human	PCL‐PANI nanofibers	Electrical	NA	Differentiation	^[^ ^228^ ^]^Garrudo, F.F.F.*, et al. Biomaterials Science* **9**, 5359–5382 (2021).
IMR‐32	In vitro/human	Multiwall carbon nanotube	Electrical	Stimulation of myelination and neurotrophic factor secretion	Proliferation	^[^ ^229^ ^]^Ghosh, S.*, et al. Acs Applied Bio Materials* **3**, 5796–5812 (2020).
HT‐22	In vitro/mouse	Multiwalled carbon nanotubes	Electrical	PI3K‐Akt pathway	Differentiation	^[^ ^230^ ^]^Ghosh, S., Roy, P. & Lahiri, D. *International Journal of Biological Macromolecules* **218**, 269–284 (2022).
HT‐22	In vitro/mouse	Carbon nanomaterials with chitosan scaffold	Electrical	Reinforce direct electrical coupliung between neurons	Proliferation and elongation morphology	^[^ ^231^ ^]^Gupta, P.*, et al. Materials Science and Engineering C‐Materials for Biological Applications* **97**, 539–551 (2019).
NSCs	In vitro/ human	Carbon nanotube	Electrical	Regulating autophagy	Differentiation, maturation, and survival	^[^ ^232^ ^]^He, L.M.*, et al. Biomaterials* **268**(2021).
ADSCs	In vitro/ human	PEG, PEDOT, PSSS microwell	Electrical	NA	Differentiation	^[^ ^233^ ^]^Heo, D.N.*, et al.Tissue Engineering Part A* **24**, 537–545 (2018).
PC12	In vitro/rat	PLLA‐PPY	Electrical	Modulation of calcium and potassium concentrations	Differentiation	^[^ ^234^ ^]^Jing, W.*, et al. Acs Chemical Neuroscience* **10**, 348–357 (2019).
NSCs	In vitro/rat	Gold nanocage‐coated glass coverslips	Laser‐induced thermal stimulation	Heat shock protein	Differentiation	^[^ ^235^ ^]^Jung, S.*, et al. Nanomaterials* **11**(2021).
NSCs	In vitro/mouse	Microfluidic microelectrode array	Electrical	NA	Differentiation	^[^ ^236^ ^]^Kim, J.W.*, et al. Lab on a Chip* **22**, 2122–2130 (2022).
NSCs	In vitro/mice	PEG hydrogel and silver nanowire composite	Electrical	NA	Differentiation	^[^ ^237^ ^]^Lee, J.M.*, et al. Sensors and Actuators B‐Chemical* **258**, 1042–1050 (2018).
NCSCs	In vitro/ human	Micropattern	Induced mechanical strain	Induced calponin‐1 (CNN1) and myosin heavy chain (MHC)	Differentiation	^[^ ^238^ ^]^Li, X.*, et al. Plos One* **6**(2011).
PC12	In vitro/rat	GElMA/GO hydrogel	Electrical	Alters cell's membrane permeability due to formation of pores	Differentiation	^[^ ^239^ ^]^Mohammadalizadeh, et al. *Nanotechnology* **33**(2022).
MSCs	In vitro/rat	Thermoplastic urethane (TPU) MWNT nanofibers	Electrical	Modulation of synaptic ion transfer	Proliferatioand differentiation	^[^ ^240^ ^]^Pouladzadeh, F., Katbab, A.A., Haghighipour, N. & Kashi, E. *European Polymer Journal* **105**, 286–296 (2018).
BMSCs	In vitro/rat	GO	Electrical	Biomaterial‐cell interaction under mechanical stress	Differentiation	^[^ ^241^ ^]^Qiao, K.*, et al. Materials Science and Engineering C‐Materials for Biological Applications* **93**, 853–863 (2018).
SH‐SY5Y	In vitro/human	GO	Electrical	Unknown	Differentiation	^[^ ^242^ ^]^Qing, H.B.*, et al*. *Acs Applied Materials & Interfaces* **10**, 39228–39237 (2018).
NSCs	In vitro/ mouse	AuNP	Circularly polarized photons	Mechanical deformation of actin nanofibers	Differentiation	^[^ ^243^ ^]^Qu, A.H.*, et al. Nature Biomedical Engineering* **5**, 103‐+ (2021).
MSCs	In vitro	PCL/CS/AuNP	Electrical	NA	Differentiation	^[^ ^244^ ^]^Rahimzadegan, M., et al. *Biomaterials Advances* **134**(2022).
NSC	In vitro/ human	PEDOT/PSS nanofiber	Electrical	NA	Differentiation	^[^ ^245^ ^]^itzau‐Reid, K.I.*, et al. Advanced Functional Materials* **30**(2020).
NSC	In vitro/mouse	CNT nanocomposite	Electrical	Activation of focal adhesion kinase	Differentiation	^[^ ^246^ ^]^Shao, H.*, et al.* 1*Biomaterials* **175**, 93–109 (2018).
ES‐derived NSCs	In vitro mouse	SEBS/PEDOT:PSS	Electrical	Modulation of actin cytoskeleton	Differentiation	^[^ ^247^ ^]^Srivastava, N.*, et al. Tissue Engineering Part A* **19**, 1984–1993 (2013).
Self powered patch	In vitro and in vivo rat	Self‐powered smart patch	Electrical and biological	CREB pathway	Nerve regeneration	^[^ ^248^ ^]^Tan, M.H.*, et al. Biomaterials* **283**(2022).
Schwann cell/PC12/ESC/	Rat/rat/ mouse/ in vitro	Aligned silk fibroin	Electrical	NA	Differentiation	^[^ ^249^ ^]^Wang, L.L.*, et al. Acs Biomaterials Science & Engineering* **5**, 613–622 (2019).
PC12	Rat/ in vitro	Poly(3‐hexylthiophene) (P3HT) microfibers	Light irradiation	Assebly of focial adhesion related proteins (i.e.vinculin and paxillin)	Differentiation	^[^ ^250^ ^]^Wu, Y.J.*, et al. Acs Applied Materials & Interfaces* **11**, 4833–4841 (2019).
Cortical cells	Rat / in vitro	Polypyrrole (PPy) coated electrospun polyacrylonitrile (PAN) nanofibers	Electrical	Modulation of calcium ion signaling	Differentiation	^[^ ^251^ ^]^Xu, Q.W., et al. Rsc Advances 8, 11027–11035 (2018).
NSCs	Rat/ in vitro	Poly‐pyrrole collagen hydrogel	Electrical	Modulation of calcium ion channels	Differentiation	^[^ ^252^ ^]^Xu, X.Z.*, et al.* *Frontiers in Bioengineering and Biotechnology* **10**(2022).
MSCs	Human/ in vitro	Polypyrrole/polylactide composite nanofiber	Electrical	NA	Differentiation	^[^ ^253^ ^]^Zhou, J.F.*, et al. Frontiers of Materials Science* **10**, 260–269 (2016).
NSCs	In vitro/ in vivo/ mouse	Biocompatible conducting polyer with tannic acid	Electrical	Restoration of interrupted spinal circuit	Differentiation	^[^ ^254^ ^]^Zhou, L.*, et al.* Soft *Acs Nano* **12**, 10957–10967 (2018).
NSC	In vitro/ mouse	CNTs	Electrical	Increased fibronectin adsorption	Differentiation	^[^ ^255^ ^]^Zhu, W.*, et al. Nanomedicine‐Nanotechnology Biology and Medicine* **14**, 2485–2494 (2018).
MSCs	In vitro/ human	Poly‐(ethylene glycol)‐diacrylate hydrogel	Ultrasound	NA	Differentiation	^[^ ^256^ ^]^Aliabouzar, M., et al. *Scientific Reports* **6**(2016).
BMSCs	In vitro/human	Alginate hydrogel	Ultrasound	RGD peptide	Differentiate	^[^ ^257^ ^]^Assanah, F., et al. *Regenerative Engineering and Translational Medicine* (2023).
PDMCs	In vitro/ human	Microfluidic device	Chemical (3‐isobutyl‐1‐methylxanthine) and mechanical shear stress	Stimulation of cytoskeleton proteins	Differentiation	^[^ ^258^ ^]^Cheng, Y.C.*, et al. fluidics and Nanofluidics* **18**, 587–598 (2015).
ESCs	In vitro/ mouse	Magnetic nanoparticle	Mechanical	Activation of PI3K/Akt and ERK1/2	Differentiation	^[^ ^259^ ^]^Du, V.*, et al. Nature Communications* **8**(2017).
Osteoclast precursor	In vitro / mouse	Cone and plate flow chamber	Shear stress	Modulation of calcium channels	Differentiation	^[^ ^260^ ^]^Gao, Y.*, et al. Biomechanics and Modeling in Mechanobiology* **18**, 1731–1744 (2019).
MSCs	In vitro/ bovine	Flow chamber	Shear stress	Integrin signaling	Differentiation	^[^ ^261^ ^]^Goldman, S.M. et al. *Bmc Biotechnology* **16**(2016).
MSCs	In vitro/ human	Bioreactor	Shear and compressive stress	NA	Differentiation	^[^ ^262^ ^]^Guo, T.*, et al*. *Annals of Biomedical Engineering* **44**, 2103–2113 (2016).
HUMSCs	In vitro/ human	Chitosan scaffold	Magnetic	NA	Differentiation	^[^ ^263^ ^]^Hao, M.Y., et al. *Bioresources and Bioprocessing* **8**(2021).
iPSCs	In vitro/ human	Collagen gel	Vibration	NA	Differentiation	^[^ ^264^ ^]^Kosawada, T., *Microsystem Technologies‐Micro‐and Nanosystems‐Information Storage and Processing Systems* **24**, 625–638 (2018).
iPSCs	In vitro/ human	Microfluidic device	Shear stress	NA	Differentiation	^[^ ^265^ ^]^Kreutzer, J.*, et al*. *Biomechanics and Modeling in Mechanobiology* **19**, 291–303 (2020).
ADMSCs	In vitro/ human	Nickel nanowires (Ni NW)	Magnetic	Modulation of ion channels	Differentiation	^[^ ^266^ ^]^Labusca, L.*, et al. Scientific Reports* **12**(2022).
MSCs	In vitro/ rat	Methacrylated hyaluronic acids	Compression	Not well understood	Differentiation	^[^ ^267^ ^]^Lin, S.*, et al. Stem Cell Research & Therapy* **8**(2017).
ADMSCs	In vitro/ human	Gelatin and pullulan electrofibers	Electrical stress	ATP increases that modulates the influx of calcium and sodium ions and cAMP.PKA signaling.	Differentiation	^[^ ^268^ ^]^Nosoudi, N.*, et al. Scientific Reports* **11**(2021).
NSCs	In vivo/ mice	AuNP	Light‐induced mechanical stress	Mechanical deformation of actin nanofibres	Differentiation	^[^ ^243^ ^]^Qu, A.H.*, et al*. *Nature Biomedical Engineering* **5**, 103‐+ (2021).
MSCs	In vitro/ Porcine	Agarose hydrogel	Dynamic compression	TGF‐β3 dependent pathway	Differentiation	^[^ ^270^ ^]^Thorpe, S.D.*, et al. Annals of Biomedical Engineering* **38**, 2896–2909 (2010).
BMSCs	In vivo/ mouse	Cyclic RGD nanobubbles	Ultrasound	Integrin signaling & TRPM7 regulation	Osteogenesis	^[^ ^271^ ^]^Yao, H.*, et al. Journal of Nanobiotechnology* **20**(2022).
Osteoblast cells	In vitro/ human	Magnetic iron oxide NP in collagen gels	Magnetic	Matrix‐cell interactions and increased expression of Runx2, ON, BMP‐2 and BMP‐4	Osteogenesis	^[^ ^272^ ^]^Yuan, Z.Y.*, et al. Scientific Reports* **8**(2018).
MSCs	In vitro/ human	3D scaffolds of RGDS peptide/hydroxyapatite NP	Ultrasound	NA	Osteogenesis	^[^ ^273^ ^]^hou, X.*, et al.Scientific Reports* **6**(2016).

### 4.4 Other Stimuli‐Responsive Biomaterials for Dynamic Mechanomodulation of Stem Cell Fates

Although light‐ and magnetic‐responsive materials have been the most explored for the remote 4D mechanomodulation of stem cell fates, there are also other types of forces convertible into mechanical forces with their own advantages. For example, ultrasound, especially low‐intensity pulsed ultrasound (also known as LIPUS) has been recently established for the mechanical modulation of stem cells. LIPUS has been developed for modulating stem cell osteogenesis,^[^ ^201^ ^]^ chondrogenesis,^[^ ^202^ ^]^ hepatogenesis,^[^ ^203^ ^]^ neurogenesis,^[^ ^108^, ^204^, ^205^ ^]^ and adipogenesis,^[^ ^206^ ^]^ through microbubble‐triggered pathways, including TWIST‐SDF, Rock‐MEK‐ERK, Wnt/β‐catenin, mTOR, and PI3K pathways. Also, thermal energies in the form of mild heating have also been used for the stimulation of microsphere expansions that trigger the mechanical activation of cells.^[^ ^207^ ^]^ Interestingly, though mechanisms remain largely unknown, electromagnetic fields have also been used for the mechanomodulation of stem cells. For example, graphene placed under a rotating magnetic field converts magnetic energy into electrical signals that induce the conversion of adipocytes into neuron‐like cells.^[^ ^208^ ^]^ Similarly, gold nanoparticle tethered with RGD can be magnetized under an alternating magnetic field (optimally at 2 × 10^−3^T/100 Hz), which further enhanced the direct reprogramming of fibroblasts into dopaminergic neurons. Gene‐set enrichment analysis and Chip‐Seq analysis seem to suggest the mechanism is mostly through Brd2‐mediated epigenetic modulation of the transdifferentiation.^[^ ^209^ ^]^ Also, chemical energies have been associated with the mechanomodulation of macrophages as well. Mg^2+^‐bisphosphonate nanoparticle with reversible intercalation of magnesium ion in response to the addition of ethylenediaminetetraacetic acid can also modulate the attachment and thus the polarization of macrophages in vitro.^[^ ^210^ ^]^ Additionally, the differentiation of bone marrow stem cells into chondrocytes,^[^ ^211^ ^]^ osteoblasts,^[^ ^212^ ^]^ and neural cells has also been directly modulated by electromagnetic fields.^[^ ^213^ ^]^ Although these physical stimuli clearly represent promising means of mechanomodulation of stem cells in a remote and dynamic manner, their detailed mechanism needs to be elucidated before their broader translation into mechanotransduction studies and clinical applications.

## 5 Future Perspectives

Building on substantial progress in mechanobiology, biomaterials with well‐defined properties such as stiffness, viscoelasticity, biodegradation rates, and integrin‐binding ligands have already shown significant clinical potential for enhancing stem cell therapies. Nevertheless, several major hurdles continue to impede the translation of mechanomodulatory biomaterials for controlling cell fate and developing effective therapies. A key challenge is that conclusions derived from in vitro studies often fail to translate directly to in vivo contexts. Current in vitro models of mechanobiology typically rely on 2D cultures combined with mechanical stimulation techniques such as engineered biomaterial substrates, optical or magnetic tweezers, and Flexcell‐based mechanical stretching. While these platforms have been instrumental in dissecting fundamental mechanotransduction mechanisms, they do not fully recapitulate the complex 3D microenvironments and dynamic cell–cell and cell–matrix interactions present in living tissues. Emerging 3D culture systems, including organoids and organ‐on‐chip models, offer more physiologically relevant alternatives, but they remain technically challenging and have yet to achieve the reproducibility and scalability required for widespread translational use. However, 2D cell culture does not recapitulate the complex cell‐cell and cell‐matrix interactions in 3D. In addition, current approaches for applying mechanical forces are limited in scale. For example, optical and magnetic tweezers could only manipulate a few cells in parallel when high accuracy is desired. However, for a mechanistic understanding of the dynamic mechanobiology processes, larger‐scale mechanical stimulation is essential. Flexcell, in contrast, could manipulate large‐scale cell forces, but its force field is heterogeneous and less accurate compared to optical tweezers. The advancement in nano/micro fabrication and instrumentation would bring new opportunities for realizing both large‐scale and precise manipulation of cell mechanotransduction. This would allow for further integration of advanced omics such as ATAC‐seq, Chip‐seq, Hi‐C, and spatial multiomics for comprehensively understanding the pathways associated with mechanotransduction.

Moreover, advances in intravital microscopy, multiphoton imaging, and light‐sheet microscopy enable real‐time, high‐resolution visualization of cellular and tissue dynamics within physiologically relevant 3D environments in living organisms, thereby enhancing the study of mechanobiology. Still, the frequently used rodent models miss critical information about genes unique in human development and diseases. Therefore, an important emerging direction is the mechanobiology studies using 3D organoid models, cially induced pluripotent stem cells (iPSCs) derived from patients. Nevertheless, the mechanical microenvironment provided by Matrigel® can be heterogeneous with tumor origin. The composition of Matrigel includes over 100 components that can be categorized into two main groups: structural ECM proteins (collagen, laminin, fibronectin) and soluble signaling molecules, particularly various growth factors. It suffers from batch‐to‐batch variations and may also induce tumorogenesis. Therefore, the potential for translating Matrigel‐derived organoid models into non‐cancerous clinical applications has been limited.

To address this limitation, significant research efforts have been directed toward engineering synthetic Matrigel substitutes with well‐defined biochemical composition and mechanical characteristics. These synthetic ECM platforms enable independent control over mechanical properties—including stiffness, viscoelasticity, and adhesive ligand presentation—facilitating systematic investigation of mechanical cue contributions to organoid development. However, the inherent complexity of 3D organoid systems, characterized by heterogeneous cell‐cell interactions and dynamically evolving signaling pathways during prolonged culture, presents ongoing challenges. Advanced omics, including spatial transcriptomics, single‐cell multiomics, and materiomics, would be helpful to elucidate the complex mechanobiological dynamics associated with organogenesis. In parallel, mechanobiological principles derived from the mechanobiological omic studies could also further guide the design of novel synthetic matrices for dynamic control over stem cell fates as well as for clinical translation of mechanotherapy.

Similarly, results from animal studies may not readily translate to treating human patients. Heterogeneity in stem cells derived from different patients further compounds this problem. A strategy to mitigate this issue is to screen biomaterials for patient‐specific stem cells before transplantation. Tailoring the materials for the target cells and further evaluating them in human disease‐like models would significantly reduce the variations from in vitro and animal studies to human patients. This also necessitates the development of more accurate human disease models, such as organoids and organ‐on‐chip systems. Personalized mechanobiology using patient‐derived iPSCs and more programmable biomaterials will also be tremendously beneficial for translating mechanotherapy into clinical applications.

Last, another major hurdle for translational mechanotherapy is the challenge associated with the effective modulation of bioscaffolds and hydrogel micro/nano‐structures in vivo. As a fundamental property for modulating stem cell mechanotransduction and differentiation, micro/nano‐structures have been intensively investigated in vitro. Advancements in microfabrication techniques have allowed us to precisely tune the substrate topographies and study their effects on mechanotransduction pathways. Although direct transplantation of biomaterial substrates featuring well‐defined micro‐ or nano‐scale structures can be beneficial for specific stem cell applications, including skin wound healing and nerve regeneration conduits, clinical settings, particularly during the early stages of disease, often favor injectable biomaterials due to their minimal invasiveness, ease of administration, and improved reproducibility. A key goal, therefore, is to achieve precise control over the micro‐ and nanoscale structures of injectable mechanomodulatory materials such as hydrogels, a challenge that remains unresolved. Attaining this level of control would not only improve reproducibility and tunability of their mechanical properties but also enhance their therapeutic efficacy, thereby accelerating the translation of mechanomodulatory biomaterials into clinical applications. Successfully addressing this limitation would substantially enhance the efficacy of future mechanotherapies by introducing an additional level of precision and tunability to the mechanical properties of injectable biomaterials. Additionally, developing advanced mechanomodulatory devices as replacements for biomaterials would be beneficial. This is because they offer less complexity by applying forces directly to patients without introducing exogenous biomaterial implants, and they also expedite regulatory review and clinical translation. One such example is the ventilator‐based mechanical stimulation device, which has been found to reduce lung fibrosis and is readily integrated into clinically used devices. However, the field of mechanotherapy is still in its infancy. Considerable and focused efforts from biologists, biomaterial scientists, mechanical engineers, and biomedical engineers will be essential to realize the full potential of this field.

## 6 Conclusion

In summary, the function of mechanobiology in regulating cell fate and informing therapeutic strategies is now widely recognized. Recent advances in emerging fields such as mechanotherapy and mechanohealth further highlight the urgent need for next‐generation mechanomodulatory biomaterials that can precisely control cellular responses. Developing such materials will be critical not only for advancing regenerative medicine but also for improving disease modeling, accelerating drug discovery, and ultimately enabling more effective clinical therapies. To this end, this review provides up‐to‐date discussions on recent advancements in mechanomodulatory biomaterials for cell fate control and therapy. Recent progress in mechanobiological pathways, smart material design, and mechano‐bioengineering over the past few years was comprehensively reviewed. First, basic concepts of the four generations of stem cell therapies, central mechanotransduction mechanisms, and key mechanical cues of interest in the context of mechanobiology were introduced. Next, we summarized knowledge on mechanobiological dynamics within various cellular organelles, which provides the context for dynamic mechanomodulatory biomaterials. In discussions on stimuli‐responsive biomaterials, we focused on recent advancements in the dynamic mechanomodulation of stem cell differentiation. These advancements introduce an additional layer of complexity to mechanobiology and enable a more accurate recapitulation of the in vivo mechanomodulatory microenvironment. Incorporating new ideas and state‐of‐the‐art technologies to address emerging challenges in mechanobiology and stem cell engineering will drive the development of innovative material systems for regenerative therapies. Beyond advancing fundamental research, these efforts are expected to accelerate clinical translation by enabling more precise control of stem cell behavior, improving the reliability of disease models, and ultimately delivering more effective and personalized treatments for patients. Thus, by reviewing the state‐of‐the‐art knowledge from multidisciplinary fields of medicine, material science, engineering, biology, and biophysics, we provided an up‐to‐date discussion on the crucial topics related to biomaterial‐based mechanomodulation and mechanotransduction in stem cells. We examined the transition of mechanobiology from fundamental principles to clinical applications in this review. By highlighting recent advances and identifying key challenges, we aim to catalyze the development of next‐generation smart biomaterials—engineered systems that dynamically engage with cellular mechanosensing machinery. Such materials are essential for unlocking novel mechanotherapies and regenerative strategies, paving the way toward improving human mechanohealth.

## Conflict of Interest

The authors declare no conflict of interest.

## References

[1] G. Gillard , O. Nicolle , T. Brugière , S. Prigent , M. Pinot , G. Michaux , Curr. Biol. 2019, 29, 1360. 30930039 10.1016/j.cub.2019.02.059

[2] C. Araya , C. Carmona‐Fontaine , J. D. W. Clarke , Dev. Dyn. 2016, 245, 580. 26933766 10.1002/dvdy.24401

[3] C. Ferrai , C. Schulte , Eur. J. Cell Biol. 2024, 103, 151417. 38729084 10.1016/j.ejcb.2024.151417

[4] T. Rozario , D. W. DeSimone , Dev. Biol. 2010, 341, 126. 19854168 10.1016/j.ydbio.2009.10.026 PMC2854274

[5] S. M. Naqvi , L. M. McNamara , Front. Bioeng. Biotechnol. 2020, 8, 597661. 33381498 10.3389/fbioe.2020.597661 PMC7767888

[6] J. H. Wang , B. P. Thampatty , Biomech. Model. Mechanobiol 2006, 5, 1. 16489478 10.1007/s10237-005-0012-z

[7] E.‐S. Kang , D.‐S. Kim , I. R. Suhito , W. Lee , I. Song , T.‐H. Kim , Biomater. Res. 2018, 22, 10. 29619243 10.1186/s40824-018-0120-3 PMC5879765

[8] L. R. Smith , S. Cho , D. E. Discher , Physiology 2017, 33, 16. 10.1152/physiol.00026.2017 PMC5866410 29212889

[9] H. Lei , Z. Pei , C. Jiang , L. I. Cheng , Exploration 2023, 3, 20220001. 37933288 10.1002/EXP.20220001 PMC10582613

[10] M. Xu , J. Liao , J. Li , Y. Shi , Z. Zhang , Y. Fu , Z. Gu , H. I. Xu , Exploration 2025, 5, 270008. 40395753 10.1002/EXP.70008 PMC12087393

[11] W. Zhang , Y. Yang , B. Cui , Curr. Opin. Solid State Mater. Sci. 2021, 25, 100873. 33364912 10.1016/j.cossms.2020.100873 PMC7751896

[12] M. G. Haugh , T. J. Vaughan , C. M. Madl , R. M. Raftery , L. M. McNamara , F. J. O'Brien , S. C. Heilshorn , Biomaterials 2018, 171, 23. 29677521 10.1016/j.biomaterials.2018.04.026 PMC5997298

[13] B. Trappmann , J. E. Gautrot , J. T. Connelly , D. G. Strange , Y. Li , M. L. Oyen , M. A. Cohen Stuart , H. Boehm , B. Li , V. Vogel , Nat. Mater. 2012, 11, 642. 22635042 10.1038/nmat3339

[14] I. A. Marozas , K. S. Anseth , J. J. Cooper‐White , Biomaterials 2019, 223, 119430. 31493696 10.1016/j.biomaterials.2019.119430 PMC6764851

[15] E. Ergir , B. Bachmann , H. Redl , G. Forte , P. Ertl , Front. Physiol. 2018, 9, 1417. 30356887 10.3389/fphys.2018.01417 PMC6190857

[16] M. W. Tibbitt , K. S. Anseth , Biotechnol. Bioeng. 2009, 103, 655. 19472329 10.1002/bit.22361 PMC2997742

[17] M. P. Nikolova , M. S. Chavali , Bioact. Mater. 2019, 4, 271. 31709311 10.1016/j.bioactmat.2019.10.005 PMC6829098

[18] J. Liu , Q. Song , W. Yin , C. Li , N. An , Y. Le , Q. Wang , Y. Feng , Y. Hu , Y. Wang , Exploration 2025, 5, 20230078. 40040827 10.1002/EXP.20230078 PMC11875452

[19] Y.‐C. Chang , J.‐W. Wu , C.‐W. Wang , C. A. Jang , Front. Mol. Biosci. 2020, 6, 157. 32118029 10.3389/fmolb.2019.00157 PMC7025494

[20] S. Li , D. Yang , L. Gao , Y. Wang , Q. Peng , Biophys. Rep. 2020, 6, 33.

[21] Y. He , S. Zhang , Y. She , Z. Liu , Y. Zhu , Q. Cheng , X. I. Ji , Exploration 2024, 4, 20230164. 39713200 10.1002/EXP.20230164 PMC11655310

[22] H. De Belly , E. K. Paluch , K. J. Chalut , Nat. Rev. Mol. Cell Biol. 2022, 23, 465. 35365816 10.1038/s41580-022-00472-z

[23] D. Mohammed , M. Versaevel , C. Bruyère , L. Alaimo , M. Luciano , E. Vercruysse , A. Procès , S. Gabriele , Front. Bioeng. Biotechnol. 2019, 7, 162. 31380357 10.3389/fbioe.2019.00162 PMC6646473

[24] F. Zanconato , M. Forcato , G. Battilana , L. Azzolin , E. Quaranta , B. Bodega , A. Rosato , S. Bicciato , M. Cordenonsi , S. Piccolo , Nat. Cell Biol. 2015, 17, 1218. 26258633 10.1038/ncb3216 PMC6186417

[25] S. Piccolo , S. Dupont , M. Cordenonsi , Physiol. Rev. 2014, 94, 1287. 25287865 10.1152/physrev.00005.2014

[26] S. Dupont , L. Morsut , M. Aragona , E. Enzo , S. Giulitti , M. Cordenonsi , F. Zanconato , J. Le Digabel , M. Forcato , S. Bicciato , N. Elvassore , S. Piccolo , Nature 2011, 474, 179. 21654799 10.1038/nature10137

[27] T. Panciera , L. Azzolin , M. Cordenonsi , S. Piccolo , Nat. Rev. Mol. Cell Biol. 2017, 18, 758. 28951564 10.1038/nrm.2017.87 PMC6192510

[28] B. Zhao , L. Li , L. Wang , C.‐Y. Wang , J. Yu , K.‐L. Guan , Genes Dev. 2012, 26, 54. 22215811 10.1101/gad.173435.111 PMC3258966

[29] M. Aragona , T. Panciera , A. Manfrin , S. Giulitti , F. Michielin , N. Elvassore , S. Dupont , S. Piccolo , Cell 2013, 154, 1047. 23954413 10.1016/j.cell.2013.07.042

[30] G. Battilana , F. Zanconato , S. Piccolo , Cell Stress 2021, 5, 167. 34782888 10.15698/cst2021.11.258 PMC8561301

[31] T. Bertero , W. M. Oldham , K. A. Cottrill , S. Pisano , R. R. Vanderpool , Q. Yu , J. Zhao , Y. Tai , Y. Tang , Y. Y. Zhang , S. Rehman , M. Sugahara , Z. Qi , J. 3rd Gorcsan , S. O. Vargas , R. Saggar , R. Saggar , W. D. Wallace , D. J. Ross , K. J. Haley , A. B. Waxman , V. N. Parikh , T. De Marco , P. Y. Hsue , A. Morris , M. A. Simon , K. A. Norris , C. Gaggioli , J. Loscalzo , J. Fessel , et al., J. Clin. Invest. 2016, 126, 3313. 27548520 10.1172/JCI86387 PMC5004943

[32] S. R. Caliari , M. Perepelyuk , B. D. Cosgrove , S. J. Tsai , G. Y. Lee , R. L. Mauck , R. G. Wells , J. A. Burdick , Sci. Rep. 2016, 6, 21387. 26906177 10.1038/srep21387 PMC4764908

[33] A. Totaro , M. Castellan , G. Battilana , F. Zanconato , L. Azzolin , S. Giulitti , M. Cordenonsi , S. Piccolo , Nat. Commun. 2017, 8, 15206. 28513598 10.1038/ncomms15206 PMC5442321

[34] B. Zhao , X. Wei , W. Li , R. S. Udan , Q. Yang , J. Kim , J. Xie , T. Ikenoue , J. Yu , L. Li , P. Zheng , K. Ye , A. Chinnaiyan , G. Halder , Z. C. Lai , K. L. Guan , Genes Dev. 2007, 21, 2747. 17974916 10.1101/gad.1602907 PMC2045129

[35] C. Hsiao , M. Lampe , S. Nillasithanukroh , W. Han , X. Lian , S. P. Palecek , Biotechnol. J. 2016, 11, 662. 26766309 10.1002/biot.201500374 PMC4850094

[36] Y. Tang , R. G. Rowe , E. L. Botvinick , A. Kurup , A. J. Putnam , M. Seiki , V. M. Weaver , E. T. Keller , S. Goldstein , J. Dai , D. Begun , T. Saunders , S. J. Weiss , Dev. Cell 2013, 25, 402. 23685250 10.1016/j.devcel.2013.04.011 PMC3736823

[37] N. Gjorevski , N. Sachs , A. Manfrin , S. Giger , M. E. Bragina , P. Ordóñez‐Morán , H. Clevers , M. P. Lutolf , Nature 2016, 539, 560. 27851739 10.1038/nature20168

[38] Y. Shi , X. Han , Z. Zhang , J. Xu , G. Liu , BMEMat 2024, 3, 12129.

[39] J. K. Hu , W. Du , S. J. Shelton , M. C. Oldham , C. M. DiPersio , O. D. Klein , Cell Stem Cell 2017, 21, 91. 28457749 10.1016/j.stem.2017.03.023 PMC5501749

[40] T. Panciera , L. Azzolin , A. Fujimura , D. Di Biagio , C. Frasson , S. Bresolin , S. Soligo , G. Basso , S. Bicciato , A. Rosato , M. Cordenonsi , S. Piccolo , Cell Stem Cell 2016, 19, 725. 27641305 10.1016/j.stem.2016.08.009 PMC5145813

[41] M. Ohgushi , M. Minaguchi , Y. Sasai , Cell Stem Cell 2015, 17, 448. 26321201 10.1016/j.stem.2015.07.009

[42] T. A. Beyer , A. Weiss , Y. Khomchuk , K. Huang , A. A. Ogunjimi , X. Varelas , J. L. Wrana , Cell Rep. 2013, 5, 1611. 24332857 10.1016/j.celrep.2013.11.021

[43] K. M. Kim , Y. J. Choi , J. H. Hwang , A. R. Kim , H. J. Cho , E. S. Hwang , J. Y. Park , S. H. Lee , J. H. Hong , PLoS One 2014, 9, 92427. 10.1371/journal.pone.0092427 PMC3962409 24658423

[44] M. Xin , Y. Kim , L. B. Sutherland , M. Murakami , X. Qi , J. McAnally , E. R. Porrello , A. I. Mahmoud , W. Tan , J. M. Shelton , J. A. Richardson , H. A. Sadek , R. Bassel‐Duby , E. N. Olson , Proc. Natl. Acad. Sci. USA 2013, 110, 13839. 23918388 10.1073/pnas.1313192110 PMC3752208

[45] D. Mosqueira , S. Pagliari , K. Uto , M. Ebara , S. Romanazzo , C. Escobedo‐Lucea , J. Nakanishi , A. Taniguchi , O. Franzese , P. Di Nardo , M. J. Goumans , E. Traversa , P. Pinto‐do‐Ó , T. Aoyagi , G. Forte , ACS Nano 2014, 8, 2033. 24483337 10.1021/nn4058984

[46] C. S. Nowell , P. D. Odermatt , L. Azzolin , S. Hohnel , E. F. Wagner , G. E. Fantner , M. P. Lutolf , Y. Barrandon , S. Piccolo , F. Radtke , Nat. Cell Biol. 2016, 18, 168. 26689676 10.1038/ncb3290 PMC6195194

[47] J. Wu , A. H. Lewis , J. Grandl , Trends Biochem. Sci. 2017, 42, 57. 27743844 10.1016/j.tibs.2016.09.004 PMC5407468

[48] B. Martinac , J. Adler , C. Kung , Nature 1990, 348, 261. 1700306 10.1038/348261a0

[49] J. Teng , S. Loukin , A. Anishkin , C. Kung , Pflügers Archiv. – Eur. J. Physiol. 2015, 467, 27. 24888690 10.1007/s00424-014-1530-2 PMC4254906

[50] J. Árnadóttir , M. Chalfie , Annu. Rev. Biophys. 2010, 39, 111. 20192782 10.1146/annurev.biophys.37.032807.125836

[51] S. Katta , M. Krieg , M. B. Goodman , Annu. Rev. Cell Dev. Biol. 2015, 31, 347. 26566115 10.1146/annurev-cellbio-100913-013426

[52] J. Richardson , A. Kotevski , K. Poole , FEBS J. 2022, 289, 4447. 34060230 10.1111/febs.16041

[53] B. Xiao , Nat. Rev. Mol. Cell Biol. 2024, 25, 886. 39251883 10.1038/s41580-024-00773-5

[54] Y. Qi , L. Andolfi , F. Frattini , F. Mayer , M. Lazzarino , J. Hu , Nat. Commun. 2015, 6, 8512. 26443885 10.1038/ncomms9512 PMC4633829

[55] J. L. Nourse , M. M. Pathak , Semin. Cell Dev. Biol. 2017, 71, 3. 28676421 10.1016/j.semcdb.2017.06.018 PMC6070642

[56] A. H. Lewis , J. Grandl , Elife 2015, 4, 12088. 10.7554/eLife.12088 PMC4718726 26646186

[57] Z. Jia , R. Ikeda , J. Ling , V. Viatchenko‐Karpinski , J. G. Gu , J. Biol. Chem. 2016, 291, 9087. 26929410 10.1074/jbc.M115.692384 PMC4861477

[58] C. A. Hartzell , K. I. Jankowska , J. K. Burkhardt , R. S. Lewis , Elife 2016, 5, 14850. 10.7554/eLife.14850 PMC4956410 27440222

[59] E. N. Olson , A. Nordheim , Nat. Rev. Mol. Cell Biol. 2010, 11, 353. 20414257 10.1038/nrm2890 PMC3073350

[60] J. Li , B. Hou , S. Tumova , K. Muraki , A. Bruns , M. J. Ludlow , A. Sedo , A. J. Hyman , L. McKeown , R. S. Young , N. Y. Yuldasheva , Y. Majeed , L. A. Wilson , B. Rode , M. A. Bailey , H. R. Kim , Z. Fu , D. A. Carter , J. Bilton , H. Imrie , P. Ajuh , T. N. Dear , R. M. Cubbon , M. T. Kearney , R. K. Prasad , P. C. Evans , J. F. Ainscough , D. J. Beech , Nature 2014, 515, 279. 25119035 10.1038/nature13701 PMC4230887

[61] D. M. Ornitz , N. Itoh , Wiley Interdiscip. Rev. Dev. Biol. 2015, 4, 215. 25772309 10.1002/wdev.176 PMC4393358

[62] L. Xiao , X. Yuan , S. J. Sharkis , Stem Cells 2006, 24, 1476. 16456129 10.1634/stemcells.2005-0299

[63] R. H. Xu , R. M. Peck , D. S. Li , X. Feng , T. Ludwig , J. A. Thomson , Nat. Methods 2005, 2, 185. 15782187 10.1038/nmeth744

[64] C. J. O'Callaghan , B. Williams , Hypertension 2000, 36, 319. 10988258 10.1161/01.hyp.36.3.319

[65] S. Saha , L. Ji , J. J. de Pablo , S. P. Palecek , Biophys. J. 2008, 94, 4123. 18234825 10.1529/biophysj.107.119891 PMC2367167

[66] K. Draganova , M. Zemke , L. Zurkirchen , T. Valenta , C. Cantù , M. Okoniewski , M. T. Schmid , R. Hoffmans , M. Götz , K. Basler , L. Sommer , Stem Cells 2015, 33, 170. 25182747 10.1002/stem.1820

[67] K. N. Dahl , A. J. S. Ribeiro , J. Lammerding , Circ. Res. 2008, 102, 1307. 18535268 10.1161/CIRCRESAHA.108.173989 PMC2717705

[68] Y. Song , J. Soto , B. Chen , T. Hoffman , W. Zhao , N. Zhu , Q. Peng , L. Liu , C. Ly , P. K. Wong , Y. Wang , A. C. Rowat , S. K. Kurdistani , S. Li , Nat. Mater. 2022, 21, 1191. 35927431 10.1038/s41563-022-01312-3 PMC9529815

[69] M. Maurer , J. Lammerding , Annu. Rev. Biomed. Eng. 2019, 21, 443. 30916994 10.1146/annurev-bioeng-060418-052139 PMC6815102

[70] C. L. Stewart , K. J. Roux , B. Burke , Science 2007, 318, 1408. 18048680 10.1126/science.1142034

[71] T. Bouzid , E. Kim , B. D. Riehl , A. M. Esfahani , J. Rosenbohm , R. Yang , B. Duan , J. Y. Lim , J. Biol. Eng. 2019, 13, 68. 31406505 10.1186/s13036-019-0197-9 PMC6686368

[72] D. A. Fletcher , R. D. Mullins , Nature 2010, 463, 485. 20110992 10.1038/nature08908 PMC2851742

[73] M. Soheilypour , M. Peyro , Z. Jahed , M. R. K. Mofrad , Cell Mol. Bioeng. 2016, 9, 217. 10.1242/jcs.184184 27530973

[74] Y. Gruenbaum , R. Foisner , Annu. Rev. Biochem. 2015, 84, 131. 25747401 10.1146/annurev-biochem-060614-034115

[75] J. Swift , I. L. Ivanovska , A. Buxboim , T. Harada , P. C. Dingal , J. Pinter , J. D. Pajerowski , K. R. Spinler , J. W. Shin , M. Tewari , F. Rehfeldt , D. W. Speicher , D. E. Discher , Science 2013, 341, 1240104. 23990565 10.1126/science.1240104 PMC3976548

[76] C. Schwartz , M. Fischer , K. Mamchaoui , A. Bigot , T. Lok , C. Verdier , A. Duperray , R. Michel , I. Holt , T. Voit , S. Quijano‐Roy , G. Bonne , C. Coirault , Sci. Rep. 2017, 7, 1253. 28455503 10.1038/s41598-017-01324-z PMC5430732

[77] M. M. Nava , Y. A. Miroshnikova , L. C. Biggs , D. B. Whitefield , F. Metge , J. Boucas , H. Vihinen , E. Jokitalo , X. Li , J. M. García Arcos , B. Hoffmann , R. Merkel , C. M. Niessen , K. N. Dahl , S. A. Wickström , Cell 2020, 181, 800. 32302590 10.1016/j.cell.2020.03.052 PMC7237863

[78] H. T. J. Gilbert , V. Mallikarjun , O. Dobre , M. R. Jackson , R. Pedley , A. P. Gilmore , S. M. Richardson , J. Swift , Nat. Commun. 2019, 10, 4149. 31515493 10.1038/s41467-019-11923-1 PMC6742657

[79] G. Uzer , G. Bas , B. Sen , Z. Xie , S. Birks , M. Olcum , C. McGrath , M. Styner , J. Rubin , J. Biomech. 2018, 74, 32. 29691054 10.1016/j.jbiomech.2018.04.013 PMC5962429

[80] E. Markiewicz , K. Tilgner , N. Barker , M. van de Wetering , H. Clevers , M. Dorobek , I. Hausmanowa‐Petrusewicz , F. C. Ramaekers , J. L. Broers , W. M. Blankesteijn , G. Salpingidou , R. G. Wilson , J. A. Ellis , C. J. Hutchison , EMBO J. 2006, 25, 3275. 16858403 10.1038/sj.emboj.7601230 PMC1523183

[81] J. Demmerle , A. J. Koch , J. M. Holaska , J. Biol. Chem. 2012, 287, 22080. 22570481 10.1074/jbc.M111.325308 PMC3381166

[82] K. Kuwahara , T. Barrientos , G. C. Pipes , S. Li , E. N. Olson , Mol. Cell. Biol. 2005, 25, 3173. 15798203 10.1128/MCB.25.8.3173-3181.2005 PMC1069631

[83] M. J. Boland , K. L. Nazor , J. F. Loring , Circ. Res. 2014, 115, 311. 24989490 10.1161/CIRCRESAHA.115.301517 PMC4229506

[84] T. Cremer , M. Cremer , Cold Spring Harb Perspect. Biol. 2010, 2, a003889. 20300217 10.1101/cshperspect.a003889 PMC2829961

[85] K. P. Eagen , Trends Biochem. Sci. 2018, 43, 469. 29685368 10.1016/j.tibs.2018.03.006 PMC6028237

[86] L. Yang , B. M. Conley , C. Rathnam , H.‐Y. Cho , T. Pongkulapa , B. Conklin , K.‐B. Lee , ACS Nano 2022, 16, 5577. 35301847 10.1021/acsnano.1c10344

[87] T. L. Downing , J. Soto , C. Morez , T. Houssin , A. Fritz , F. Yuan , J. Chu , S. Patel , D. V. Schaffer , S. Li , Nat. Mater. 2013, 12, 1154. 24141451 10.1038/nmat3777 PMC9675045

[88] J. Le Beyec , R. Xu , S.‐Y. Lee , C. M. Nelson , A. Rizki , J. Alcaraz , M. J. Bissell , Exp. Cell Res. 2007, 313, 3066. 17524393 10.1016/j.yexcr.2007.04.022 PMC2040058

[89] S. Huang , S. Zhao , H. Zhao , M. Wen , Z. I. Guo , Exploration 2025, 5, 20240045. 10.1002/EXP.20240045 PMC12380062 40873635

[90] K. H. Nakayama , L. Hou , N. F. Huang , Adv. Healthcare Mater. 2014, 3, 628. 10.1002/adhm.201300620 PMC4031033 24443420

[91] G. S. Hussey , J. L. Dziki , S. F. Badylak , Nat. Rev. Mater. 2018, 3, 159.

[92] A. Naba , Nat. Rev. Mol. Cell Biol. 2024, 25, 865. 39223427 10.1038/s41580-024-00767-3 PMC11931590

[93] A. M. Rosales , K. S. Anseth , Nat. Rev. Mater. 2016, 1, 1. 10.1038/natrevmats.2015.12 PMC5714327 29214058

[94] Y. Shou , X. Y. Teo , K. Z. Wu , B. Bai , A. R. Kumar , J. Low , Z. Le , A. Tay , Adv. Sci. 2023, 10, 2300670. 10.1002/advs.202300670 PMC10375194 37119518

[95] M. Risteli , H. Ruotsalainen , A. M. Salo , R. Sormunen , L. Sipilä , N. L. Baker , S. R. Lamandé , L. Vimpari‐Kauppinen , R. Myllylä , J. Biol. Chem. 2009, 284, 28204. 19696018 10.1074/jbc.M109.038190 PMC2788872

[96] A. D. Theocharis , S. S. Skandalis , C. Gialeli , N. K. Karamanos , Adv. Drug Delivery Rev. 2016, 97, 4. 10.1016/j.addr.2015.11.001 26562801

[97] A. D. Doyle , S. S. Nazari , K. M. Yamada , Phys. Biol. 2022, 19, 021002. 10.1088/1478-3975/ac4390 PMC8855216 34911051

[98] O. Chaudhuri , J. Cooper‐White , P. A. Janmey , D. J. Mooney , V. B. Shenoy , Nature 2020, 584, 535. 32848221 10.1038/s41586-020-2612-2 PMC7676152

[99] Y. Peng , Y. Zhuang , Y. Liu , H. Le , D. Li , M. Zhang , K. Liu , Y. Zhang , J. Zuo , J. I. Ding , Exploration 2023, 3, 20210043. 37933242 10.1002/EXP.20210043 PMC10624381

[100] Y. Feng , Z. Zhang , W. Tang , Y. Dai , Exploration 2023, 3, 20220173. 37933278 10.1002/EXP.20220173 PMC10582614

[101] B. Slater , J. Li , D. Indana , Y. Xie , O. Chaudhuri , T. Kim , Soft Matter 2021, 17, 10274. 34137758 10.1039/d0sm01911a PMC8695121

[102] D. Pally , A. Naba , Curr. Opin. Cell Biol. 2024, 86, 102309. 38183892 10.1016/j.ceb.2023.102309 PMC10922734

[103] H. T. McMahon , J. L. Gallop , Nature 2005, 438, 590. 16319878 10.1038/nature04396

[104] E. Sezgin , I. Levental , S. Mayor , C. Eggeling , Nat. Rev. Mol. Cell Biol. 2017, 18, 361. 28356571 10.1038/nrm.2017.16 PMC5500228

[105] Y. Ma , S. Li , X. Lin , Y. I. Chen , Exploration 2024, 4, 20230147. 39713203 10.1002/EXP.20230147 PMC11655307

[106] D. Lichtenberg , F. M. Goñi , H. Heerklotz , Trends Biochem. Sci. 2005, 30, 430. 15996869 10.1016/j.tibs.2005.06.004

[107] Z. Belhadj , Y. Qie , R. P. Carney , Y. Li , G. Nie , BMEMat 2023, 1, 12018.

[108] J. Zhao , Z. Chen , S. Liu , P. Li , S. Yu , D. Ling , F. Li , BMEMat 2024, 2, 12066.

[109] Y. Sun , Y. Hu , Y. Geng , C. Wan , Y. Liu , Y. Liao , X. Shi , J. F. Lovell , K. Yang , H. Jin , Exploration 2024, 4, 20220170. 39439494 10.1002/EXP.20220170 PMC11491297

[110] A. Saraswathibhatla , D. Indana , O. Chaudhuri , Nat. Rev. Mol. Cell Biol. 2023, 24, 495. 36849594 10.1038/s41580-023-00583-1 PMC10656994

[111] T.‐Y. Yoon , C. Jeong , S.‐W. Lee , J. H. Kim , M. C. Choi , S.‐J. Kim , M. W. Kim , S.‐D. Lee , Nat. Mater. 2006, 5, 281. 16565710 10.1038/nmat1618

[112] Y. Arinaminpathy , E. Khurana , D. M. Engelman , M. B. Gerstein , Drug Discovery Today 2009, 14, 1130. 19733256 10.1016/j.drudis.2009.08.006 PMC2796609

[113] M. B. Goodman , E. S. Haswell , V. Vásquez , J. Gen. Physiol. 2023, 155, 202213248. 10.1085/jgp.202213248 PMC9930137 36696153

[114] Y.‐C. Lin , Y. R. Guo , A. Miyagi , J. Levring , R. MacKinnon , S. Scheuring , Nature 2019, 573, 230. 31435018 10.1038/s41586-019-1499-2 PMC7258172

[115] T. Banerjee , D. Biswas , D. S. Pal , Y. Miao , P. A. Iglesias , P. N. Devreotes , Nat. Cell Biol. 2022, 24, 1499. 36202973 10.1038/s41556-022-00997-7 PMC10029748

[116] T. Banerjee , S. Matsuoka , D. Biswas , Y. Miao , D. S. Pal , Y. Kamimura , M. Ueda , P. N. Devreotes , P. A. Iglesias , Nat. Commun. 2023, 14, 7909. 38036511 10.1038/s41467-023-43615-2 PMC10689845

[117] T. Banerjee , S. Matsuoka , D. Biswas , Y. Miao , D. S. Pal , Y. Kamimura , M. Ueda , P. N. Devreotes , P. A. Iglesias , Biophys. J. 2024, 123, 484a.

[118] J. D. Humphrey , E. R. Dufresne , M. A. Schwartz , Nat. Rev. Mol. Cell Biol. 2014, 15, 802. 25355505 10.1038/nrm3896 PMC4513363

[119] O. Thoumine , P. Kocian , A. Kottelat , J.‐J. Meister , Eur. Biophys. J. 2000, 29, 398. 11081401 10.1007/s002490000087

[120] S. I. Fraley , Y. Feng , R. Krishnamurthy , D.‐H. Kim , A. Celedon , G. D. Longmore , D. Wirtz , Nat. Cell Biol. 2010, 12, 598. 20473295 10.1038/ncb2062 PMC3116660

[121] L. Moldovan , C. H. Song , Y. C. Chen , H. J. Wang , L. A. I. Ju , Exploration 2023, 3, 20230004. 37933233 10.1002/EXP.20230004 PMC10624387

[122] P. Jiang , Y. Dai , Y. Hou , J. Stein , S. S. Lin , C. Zhou , Y. Hou , R. Zhu , K. B. Lee , L. Yang , BMEMat 2025, 35, 45, 70004.

[123] S. A. Gudipaty , J. Lindblom , P. D. Loftus , M. J. Redd , K. Edes , C. F. Davey , V. Krishnegowda , J. Rosenblatt , Nature 2017, 543, 118. 28199303 10.1038/nature21407 PMC5334365

[124] L. Liu , P. He , Y. Wang , F. Ma , D. Li , Z. Bai , Y. Qu , L. Zhu , C. W. Yoon , X. Yu , Cell 2025, 188, 2621. 40179881 10.1016/j.cell.2025.02.035 PMC12085297

[125] J. Liu , C. Zhao , X. Xiao , A. Li , Y. Liu , J. Zhao , L. Fan , Z. Liang , W. Pang , W. Yao , W. Li , J. Zhou , Nat. Commun. 2023, 14, 6457. 37833282 10.1038/s41467-023-42341-z PMC10576099

[126] Y. Zhu , F. Xiao , Y. Wang , Y. Wang , J. Li , D. Zhong , Z. Huang , M. Yu , Z. Wang , J. Barbara , C. Plunkett , M. Zeng , Y. Song , T. Tan , R. Zhang , K. Xu , Z. Wang , C. Cai , X. Guan , S. Hammack , L. Zhang , Z. Shi , F. Xiang , F. Shao , J. Xu , Nature 2025, 644, E38. 40770109 10.1038/s41586-025-09444-7 PMC12367520

[127] S. Bisi , A. Disanza , C. Malinverno , E. Frittoli , A. Palamidessi , G. Scita , Curr. Opin. Cell Biol. 2013, 25, 565. 23639310 10.1016/j.ceb.2013.04.001

[128] Z. Sun , S. S. Guo , R. Fässler , J. Cell Biol. 2016, 215, 445. 27872252 10.1083/jcb.201609037 PMC5119943

[129] W. Yu , A. M. Shewan , P. Brakeman , D. J. Eastburn , A. Datta , D. M. Bryant , Q. W. Fan , W. A. Weiss , M. M. Zegers , K. E. Mostov , EMBO Rep. 2008, 9, 923. 18660750 10.1038/embor.2008.135 PMC2529350

[130] K. E. Scott , S. I. Fraley , P. Rangamani , Proc. Natl. Acad. Sci. USA 2021, 118, 2021571118. 10.1073/pnas.2021571118 PMC8157940 33990464

[131] L. Schaedel , K. John , J. Gaillard , M. V. Nachury , L. Blanchoin , M. Théry , Nat. Mater. 2015, 14, 1156. 26343914 10.1038/nmat4396 PMC4620915

[132] M. G. Mendez , D. Restle , P. A. Janmey , Biophys. J. 2014, 107, 314. 25028873 10.1016/j.bpj.2014.04.050 PMC4104054

[133] C. Östlund , E. S. Folker , J. C. Choi , E. R. Gomes , G. G. Gundersen , H. J. Worman , J. Cell Sci. 2009, 122, 4099. 19843581 10.1242/jcs.057075 PMC2776502

[134] C. Uhler , G. V. Shivashankar , Nat. Rev. Mol. Cell Biol. 2017, 18, 717. 29044247 10.1038/nrm.2017.101

[135] S. Nemec , K. A. Kilian , Nat. Rev. Mater. 2021, 6, 69.

[136] T. Yu , L. Zhang , X. Dou , R. Bai , H. Wang , J. Deng , Y. Zhang , Q. Sun , Q. Li , X. Wang , B. Han , Adv. Sci. 2022, 9, 2203734. 10.1002/advs.202203734 PMC9661832 36161289

[137] W. Xie , X. Wei , H. Kang , H. Jiang , Z. Chu , Y. Lin , Y. Hou , Q. Wei , Adv. Sci. 2023, 10, 2204594. 10.1002/advs.202204594 PMC10037983 36658771

[138] M. Hieda , T. Matsumoto , M. Isobe , S. Kurono , K. Yuka , S. Kametaka , J.‐Y. Wang , Y.‐H. Chi , K. Kameda , H. Kimura , N. Matsuura , S. Matsuura , Sci. Rep. 2021, 11, 5358. 33686165 10.1038/s41598-021-84750-4 PMC7940470

[139] J. Sun , Y. Liu , J. Sun , J. Ding , X. I. Chen , Exploration 2025, 5, 20240229. 10.1002/EXP.20240229 PMC12380060 40873640

[140] Z. Liao , T. Liu , Z. Yao , T. Hu , X. Ji , B. Yao , Exploration 2025, 5, 20230133. 40040822 10.1002/EXP.20230133 PMC11875454

[141] E. Bartolák‐Suki , J. Imsirovic , H. Parameswaran , T. J. Wellman , N. Martinez , P. G. Allen , U. Frey , B. Suki , Nat. Mater. 2015, 14, 1049. 26213900 10.1038/nmat4358

[142] S. Phuyal , P. Romani , S. Dupont , H. Farhan , Trends Cell Biol. 2023, 33, 1049. 37236902 10.1016/j.tcb.2023.05.001

[143] P. Romani , G. Benedetti , M. Cusan , M. Arboit , C. Cirillo , X. Wu , G. Rouni , V. Kostourou , M. Aragona , C. Giampietro , P. Grumati , G. Martello , S. Dupont , Nat. Cell Biol. 2024, 26, 2046. 39433949 10.1038/s41556-024-01527-3 PMC11628398

[144] P. Romani , N. Nirchio , M. Arboit , V. Barbieri , A. Tosi , F. Michielin , S. Shibuya , T. Benoist , D. Wu , C. C. T. Hindmarch , M. Giomo , A. Urciuolo , F. Giamogante , A. Roveri , P. Chakravarty , M. Montagner , T. Calì , N. Elvassore , S. L. Archer , P. De Coppi , A. Rosato , G. Martello , S. Dupont , Nat. Cell Biol. 2022, 24, 168. 35165418 10.1038/s41556-022-00843-w PMC7615745

[145] K. Qian , S. Gao , Z. Jiang , Q. Ding , Z. I. Cheng , Exploration 2024, 4, 20230063. 39175881 10.1002/EXP.20230063 PMC11335472

[146] Y. Song , Z. Zhao , L. Xu , P. Huang , J. Gao , J. Li , X. Wang , Y. Zhou , J. Wang , W. Zhao , L. Wang , C. Zheng , B. Gao , L. Jiang , K. Liu , Y. Guo , X. Yao , L. Duan , Dev. Cell 2024, 59, 1396. 38569547 10.1016/j.devcel.2024.03.014

[147] J. Townson , C. Progida , J. Cell Sci. 2025, 138, JCS263503. 39976266 10.1242/jcs.263503

[148] Y. Yang , L. A. Valencia , C.‐H. Lu , M. L. Nakamoto , C.‐T. Tsai , C. Liu , H. Yang , W. Zhang , Z. Jahed , W.‐R. Lee , F. Santoro , J. Liou , J. C. Wu , B. Cui , Nat. Cell Biol. 2024, 26, 1878. 39289582 10.1038/s41556-024-01511-x PMC11567891

[149] Y. Ravichandran , B. Goud , J.‐B. Manneville , Curr. Opin. Cell Biol. 2020, 62, 104. 31751898 10.1016/j.ceb.2019.10.003

[150] B. Cevher‐Keskin , Int. J. Mol. Sci. 2013, 14, 18181. 24013371 10.3390/ijms140918181 PMC3794775

[151] L. Fougère , M. Grison , P. Laquel , M. Montrazi , F. Cordelières , M. Fernández‐Monreal , C. Poujol , T. Uemura , A. Nakano , Y. Ito , Y. Boutté , Nat. Cell Biol. 2025, 27, 424. 40000850 10.1038/s41556-025-01624-x

[152] H. Liu , J. F. Usprech , P. K. Parameshwar , Y. Sun , C. A. Simmons , Sci. Adv. 2021, 7, abe7204. 10.1126/sciadv.abe7204 PMC8104874 33962940

[153] A. E. M. Beedle , P. Roca‐Cusachs , Curr. Opin. Cell Biol. 2023, 84, 102229. 37633090 10.1016/j.ceb.2023.102229

[154] Z. Cao , J. Liu , X. I. Yang , Exploration 2024, 4, 20230037. 39439489 10.1002/EXP.20230037 PMC11491306

[155] H. Liu , X. Liu , H. Jiang , X. I. Wang , Exploration 2025, 5, 20240182. 40585774 10.1002/EXP.20240182 PMC12199438

[156] B. Sui , T. Ding , X. Wan , Y. Chen , X. Zhang , Y. Cui , J. Pan , L. Li , X. I. Liu , Exploration 2024, 4, 20230149. 39713207 10.1002/EXP.20230149 PMC11657998

[157] S. Wang , L. Du , G. H. Peng , Cell Biosci. 2019, 9, 73. 31497278 10.1186/s13578-019-0335-6 PMC6719367

[158] C. Bao , L. Zhu , Q. Lin , H. Tian , Adv. Mater. 2015, 27, 1647. 25655424 10.1002/adma.201403783

[159] A. Romano , I. Roppolo , E. Rossegger , S. Schlögl , M. Sangermano , Materials (Basel) 2020, 13, 2777. 32575481 10.3390/ma13122777 PMC7344511

[160] M. H. Lee , Z. Yang , C. W. Lim , Y. H. Lee , S. Dongbang , C. Kang , J. S. Kim , Chem. Rev. 2013, 113, 5071. 23577659 10.1021/cr300358b

[161] Q. Lin , C. Bao , S. Cheng , Y. Yang , W. Ji , L. Zhu , J. Am. Chem. Soc. 2012, 134, 5052. 22394079 10.1021/ja300475k

[162] M. Chen , M. Zhong , J. A. Johnson , Chem. Rev. 2016, 116, 10167. 26978484 10.1021/acs.chemrev.5b00671

[163] J. C. Grim , T. E. Brown , B. A. Aguado , D. A. Chapnick , A. L. Viert , X. Liu , K. S. Anseth , ACS Cent. Sci. 2018, 4, 909. 30062120 10.1021/acscentsci.8b00325 PMC6062832

[164] D. K. Kölmel , E. T. Kool , Chem. Rev. 2017, 117, 10358. 28640998 10.1021/acs.chemrev.7b00090 PMC5580355

[165] B. D. Polizzotti , B. D. Fairbanks , K. S. Anseth , Biomacromolecules 2008, 9, 1084. 18351741 10.1021/bm7012636

[166] X. Zhang , W. Xi , C. Wang , M. Podgórski , C. N. Bowman , ACS Macro Lett. 2016, 5, 229. 28018752 10.1021/acsmacrolett.5b00923 PMC5176105

[167] C. D. Spicer , E. T. Pashuck , M. M. Stevens , Chem. Rev. 2018, 118, 7702. 30040387 10.1021/acs.chemrev.8b00253 PMC6107854

[168] C. Li , A. Iscen , L. C. Palmer , G. C. Schatz , S. I. Stupp , J. Am. Chem. Soc. 2020, 142, 8447. 32330027 10.1021/jacs.0c02201

[169] J. Sun , F. Wang , H. Zhang , K. Liu , Adv. NanoBiomed Res. 2021, 1, 2100020.

[170] E. Blasco , M. Piñol , L. Oriol , Macromol. Rapid Commun. 2014, 35, 1090. 24706548 10.1002/marc.201400007

[171] S. H. Moon , H. J. Hwang , H. R. Jeon , S. J. Park , I. S. Bae , Y. J. Yang , Front. Bioeng. Biotechnol. 2023, 11, 1127757. 36970625 10.3389/fbioe.2023.1127757 PMC10037533

[172] V. X. Truong , F. Li , J. S. Forsythe , ACS Macro Lett. 2017, 6, 657. 35650867 10.1021/acsmacrolett.7b00312

[173] P. M. Kharkar , M. S. Rehmann , K. M. Skeens , E. Maverakis , A. M. Kloxin , ACS Biomater. Sci. Eng. 2016, 2, 165. 28361125 10.1021/acsbiomaterials.5b00420 PMC5369354

[174] C. A. DeForest , B. D. Polizzotti , K. S. Anseth , Nat. Mater. 2009, 8, 659. 19543279 10.1038/nmat2473 PMC2715445

[175] A. M. Kloxin , A. M. Kasko , C. N. Salinas , K. S. Anseth , Science 2009, 324, 59. 19342581 10.1126/science.1169494 PMC2756032

[176] C. A. DeForest , D. A. Tirrell , Nat. Mater. 2015, 14, 523. 25707020 10.1038/nmat4219

[177] F. M. Andreopoulos , E. J. Beckman , A. J. Russell , Biomaterials 1998, 19, 1343. 9758034 10.1016/s0142-9612(97)00219-6

[178] S. H. Kim , Y. Sun , J. A. Kaplan , M. W. Grinstaff , J. R. Parquette , New J. Chem. 2015, 39, 3225.

[179] T. K. Claus , S. Telitel , A. Welle , M. Bastmeyer , A. P. Vogt , G. Delaittre , C. Barner‐Kowollik , Chem. Commun. (Camb) 2017, 53, 1599. 28106193 10.1039/c6cc09897e

[180] A. M. Rosales , K. M. Mabry , E. M. Nehls , K. S. Anseth , Biomacromolecules 2015, 16, 798. 25629423 10.1021/bm501710e PMC4547937

[181] W. Wiltschko , R. Wiltschko , J. Comp. Physiol., A 2005, 191, 675. 10.1007/s00359-005-0627-7 15886990

[182] A. Farzin , S. A. Etesami , J. Quint , A. Memic , A. Tamayol , Adv. Healthcare Mater. 2020, 9, 1901058. 10.1002/adhm.201901058 PMC7482193 32196144

[183] S. C. McBain , H. H. Yiu , J. Dobson , Int. J. Nanomed. 2008, 3, 169. 10.2147/ijn.s1608 PMC2527670 18686777

[184] R. Chen , G. Romero , M. G. Christiansen , A. Mohr , P. Anikeeva , Science 2015, 347, 1477. 25765068 10.1126/science.1261821

[185] J. Lee , W. Shin , Y. Lim , J. Kim , W. R. Kim , H. Kim , J.‐H. Lee , J. Cheon , Nat. Mater. 2021, 20, 1029. 33510447 10.1038/s41563-020-00896-y

[186] L. Labusca , D.‐D. Herea , C.‐M. Danceanu , A. E. Minuti , C. Stavila , M. Grigoras , D. Gherca , G. Stoian , G. Ababei , H. Chiriac , N. Lupu , Mater. Sci. Eng., C 2020, 109, 110652. 10.1016/j.msec.2020.110652 32228923

[187] B. Hu , J. Dobson , A. J. El Haj , Nanomed.: Nanotechnol. Biol. Med. 2014, 10, 45.

[188] D. Fayol , G. Frasca , C. Le Visage , F. Gazeau , N. Luciani , C. Wilhelm , Adv. Mater. 2013, 25, 2611. 23526452 10.1002/adma.201300342

[189] J. R. Henstock , M. Rotherham , H. Rashidi , K. M. Shakesheff , A. J. El Haj , Stem Cells Transl. Med. 2014, 3, 1363. 25246698 10.5966/sctm.2014-0017 PMC4214839

[190] M. Song , J. Kim , H. Shin , Y. Kim , H. Jang , Y. Park , S.‐J. Kim , Nanomaterials 2020, 10, 1684. 32867131 10.3390/nano10091684 PMC7557977

[191] C. Bertet , L. Sulak , T. Lecuit , Nature 2004, 429, 667. 15190355 10.1038/nature02590

[192] B. D. Cosgrove , K. L. Mui , T. P. Driscoll , S. R. Caliari , K. D. Mehta , R. K. Assoian , J. A. Burdick , R. L. Mauck , Nat. Mater. 2016, 15, 1297. 27525568 10.1038/nmat4725 PMC5121068

[193] Y. Lin , Y. Zou , J. Yang , Z. Han , X. Guo , X. Gao , J. Xu , Z. Gong , R. Li , Z. Li , BMEMat 2025, 3, 12133.

[194] Y. Yoshii , T. Matsuzaki , H. Ishida , S. Ihara , Dev., Growth Differ. 2005, 47, 563. 16287487 10.1111/j.1440-169X.2005.00831.x

[195] J. M. Sawyer , J. R. Harrell , G. Shemer , J. Sullivan‐Brown , M. Roh‐Johnson , B. Goldstein , Dev. Biol. 2010, 341, 5. 19751720 10.1016/j.ydbio.2009.09.009 PMC2875788

[196] C. J. Chan , M. Costanzo , T. Ruiz‐Herrero , G. Mönke , R. J. Petrie , M. Bergert , A. Diz‐Muñoz , L. Mahadevan , T. Hiiragi , Nature 2019, 571, 112. 31189957 10.1038/s41586-019-1309-x

[197] M. Maimets , M. T. Pedersen , J. Guiu , J. Dreier , M. Thodberg , Y. Antoku , P. J. Schweiger , L. Rib , R. B. Bressan , Y. Miao , K. C. Garcia , A. Sandelin , P. Serup , K. B. Jensen , Nat. Commun. 2022, 13, 715. 35132078 10.1038/s41467-022-28369-7 PMC8821716

[198] Y. H. Tan , K. C. Wang , I. L. Chin , R. W. Sanderson , J. Li , B. F. Kennedy , P. B. Noble , Y. S. Choi , Adv. Healthcare Mater. 2024, 13, 2304254. 10.1002/adhm.202304254 38593989

[199] M. Levin , Mech. Dev. 2005, 122, 3. 15582774 10.1016/j.mod.2004.08.006

[200] I. Bedzhov , S. J. Graham , C. Y. Leung , M. Zernicka‐Goetz , Philos. Trans. R Soc. Lond. B Biol. Sci. 2014, 369, 20130538. 25349447 10.1098/rstb.2013.0538 PMC4216461

[201] J. Kusuyama , K. Bandow , M. Shamoto , K. Kakimoto , T. Ohnishi , T. Matsuguchi , J. Biol. Chem. 2014, 289, 10330. 24550383 10.1074/jbc.M113.546382 PMC4036157

[202] X. Wang , Q. Lin , T. Zhang , X. Wang , K. Cheng , M. Gao , P. Xia , X. Li , Stem Cell Res. Ther. 2019, 10, 41. 30670079 10.1186/s13287-019-1142-z PMC6343259

[203] F. Li , Y. Liu , Y. Cai , X. Li , M. Bai , T. Sun , L. Du , Ultrasound Med. Biol. 2018, 44, 1044. 29499919 10.1016/j.ultrasmedbio.2018.01.005

[204] T. El‐Bialy , A. Alhadlaq , B. Wong , C. Kucharski , Ann. Biomed. Eng. 2014, 42, 1406. 24752635 10.1007/s10439-014-1013-9

[205] Y. Wu , Q. Gao , S. Zhu , Q. Wu , R. Zhu , H. Zhong , C. Xing , H. Qu , D. Wang , B. Li , G. Ning , S. Feng , Biochem. Biophys. Res. Commun. 2020, 526, 793. 32268957 10.1016/j.bbrc.2020.03.142

[206] N. Fu , X. Yang , K. Ba , Y. Fu , X. Wei , Y. Yue , G. Li , Y. Yao , J. Chen , X. Cai , C. Liang , Y. Ge , Y. Lin , Cell Proliferation 2013, 46, 312. 23692089 10.1111/cpr.12031 PMC6496813

[207] E. R. Zynda , M. J. Grimm , M. Yuan , L. Zhong , T. A. Mace , M. Capitano , J. R. Ostberg , K. P. Lee , A. Pralle , E. A. Repasky , Cell Cycle 2015, 14, 2340. 26131730 10.1080/15384101.2015.1049782 PMC4615065

[208] Z. Guo , C. Sun , H. Yang , H. Gao , N. Liang , J. Wang , S. Hu , N. Ren , J. Pang , J. Wang , N. Meng , L. Han , H. Liu , Adv. Sci. (Weinh) 2022, 9, 2104424. 35152569 10.1002/advs.202104424 PMC9109060

[209] J. Yoo , E. Lee , H. Y. Kim , D. Youn , J. Jung , H. Kim , Y. Chang , W. Lee , J. Shin , S. Baek , W. Jang , W. Jun , S. Kim , J. Hong , H.‐J. Park , C. J. Lengner , S. H. Moh , Y. Kwon , J. Kim , Nat. Nanotechnol. 2017, 12, 1006. 28737745 10.1038/nnano.2017.133

[210] H. Kang , B. Yang , K. Zhang , Q. Pan , W. Yuan , G. Li , L. Bian , Nat. Commun. 2019, 10, 1696. 30979900 10.1038/s41467-019-09733-6 PMC6461616

[211] S. Mayer‐Wagner , A. Passberger , B. Sievers , J. Aigner , B. Summer , T. S. Schiergens , V. Jansson , P. E. Müller , Bioelectromagnetics 2011, 32, 283. 21452358 10.1002/bem.20633

[212] L. Y. Sun , D. K. Hsieh , P. C. Lin , H. T. Chiu , T. W. Chiou , Bioelectromagnetics 2010, 31, 209. 19866474 10.1002/bem.20550

[213] H. Cho , Y.‐K. Seo , H.‐H. Yoon , S.‐C. Kim , S.‐M. Kim , K.‐Y. Song , J.‐K. Park , Biotechnol. Prog. 2012, 28, 1329. 22848041 10.1002/btpr.1607

[214] Z. E. Abdar , R. Jafari , P. Mohammadi , S. Nadri , International Journal of Artificial Organs 2022, 45, 695. 35773946 10.1177/03913988221109618

[215] G. Agarwal , N. Kumar , A. Srivastava , Materials Science & Engineering C‐Materials for Biological Applications 2021, 118. 10.1016/j.msec.2020.111518 33255073

[216] S. A. Cervantes , et al., Materials Science and Engineering C‐Materials for Biological Applications 2017, 79, 315. 10.1016/j.msec.2017.05.055 28629024

[217] A. Babaie , B. Bakhshandeh , A. Abedi , J. Mohammadnejad , I. Shabani , A. Ardeshirylajimi , S. R. Moosavi , J. Amini , L. Tayebi , Eur. Polym. J. 2020, 140, 110051.

[218] A. Bonisoli , A. Marino , G. Ciofani , F. Greco , Macromol. Biosci. 2017, 17, 1700128. 10.1002/mabi.201700128 28815971

[219] R. Borah , J. M. Das , J. Upadhyay , ACS Appl. Bio Mater. 2022, 5, 3193. 10.1021/acsabm.2c00171 35775198

[220] Y. Bu , H. X. Xu , X. Li , W. J. Xu , Y. X. Yin , H. L. Dai , X. B. Wang , Z. J. Huang , P. H. Xu , RSC Adv. 2018, 8, 10806. 35541536 10.1039/c8ra01059e PMC9078905

[221] H. F. Chang , S. E. Chou , J. Y. Cheng , Jove‐Journal of Visualized Experiments 2021, 170, 61917. 10.3791/61917 33938879

[222] W. F. Chang , Y. M. Hwu , J. Xu , C. J. Lin , S. W. Wang , A. S. Cheng , J. Lu , C. H. Lu , L. Y. Sung , PLoS One 2016, 11, 0165715. 10.1371/journal.pone.0165715 PMC5089679 27802323

[223] J. Chen , M. Yu , B. Gu , P. X. Ma , Z. Yin , J. Colloid Interface Sci. 2018, 514, 517. 29289734 10.1016/j.jcis.2017.12.062

[224] P. Chen , C. Xu , P. Wu , K. Liu , F. Chen , Y. Chen , H. Dai , Z. Luo , ACS Nano 2022, 16, 16513. 36174221 10.1021/acsnano.2c05818

[225] Y. K. Choi , D. H. Lee , Y. K. Seo , H. Jung , J. K. Park , H. Cho , Appl. Biochem. Biotechnol. 2014, 174, 1233. 25099373 10.1007/s12010-014-1091-z

[226] S. R. Das , M. Uz , S. Ding , M. T. Lentner , J. A. Hondred , A. A. Cargill , D. S. Sakaguchi , S. Mallapragada , J. C. Claussen , Adv. Healthcare Mater. 2017, 6, 1601087. 10.1002/adhm.201601087 28218474

[227] C. Fu , S. Pan , Y. Ma , W. Kong , Zhiping, Qi , X. Yang , Artificial Cells Nanomedicine and Biotechnology 2019, 47, 1867. 31076002 10.1080/21691401.2019.1613422

[228] F. F. F. Garrudo , et al., Biomater. Sci. 2021, 9, 5359. 34223566 10.1039/d1bm00503k

[229] S. Ghosh , S. Haldar , S. Gupta , A. Bisht , S. Chauhan , V. Kumar , P. Roy , D. Lahiri , CS Applied Bio Materials 2020, 3, 5796. 10.1021/acsabm.0c00534 35021810

[230] S. Ghos , P. Roy , D. Lahiri , Int. J. Biol. Macromol. 2022, 218, 269. 35843399 10.1016/j.ijbiomac.2022.07.087

[231] P. Gupta , et al., Materials Science and Engineering C‐Materials for Biological Applications 2019, 97, 539. 10.1016/j.msec.2018.12.065 30678940

[232] L. M. He , et al., Biomaterials 2021, 268.

[233] D. N. Heo , N. Acquah , J. Kim , S. J. Lee , N. J. Castro , L. G. Zhang , Tissue Eng., Part A 2018, 24, 537. 28741412 10.1089/ten.TEA.2017.0150

[234] W. Jing , et al., ACS Chem. Neurosci. 2019, 10, 348. 30212623 10.1021/acschemneuro.8b00286

[235] S. Jung , et al., Nanomaterials 2021, 11.35009960

[236] J. W. Kim , Y. Y. Choi , S. H. Park , J. H. Ha , H. U. Lee , T. Kang , W. Sun , B. G. Chung , Lab Chip 2022, 22, 2122. 35388823 10.1039/d1lc01158h

[237] J. M. Lee , J. Y. Moon , T. H. Kim , S. W. Lee , C. D. Ahrberg , B. G. Chung , Sens. Actuators, B 2018, 258, 1042.

[238] X. Li , Y. Yang , F. Zhou , Y. Zhang , H. Lu , Q. Jin , L. Gao , PLoS One 2011, 6, 15831. 10.1371/journal.pone.0015831 PMC3026788 21283567

[239] M. Mohammadalizadeh , S. Dabirian , M. Akrami , Z. Hesari , Nanotechnology 2022, 33, 375101. 10.1088/1361-6528/ac7402 35623211

[240] F. Pouladzadeh , A. A. Katbab , N. Haghighipour , E. Kashi , Eur. Polym. J. 2018, 105, 286.

[241] K. Qiao , et al., Materials Science and Engineering C‐Materials for Biological Applications 2018, 93, 853. 10.1016/j.msec.2018.08.047 30274121

[242] H. B. Qing , G. Jin , G. Zhao , Y. Ma , X. Zhang , B. Sha , Z. Luo , T. J. Lu , F. Xu , ACS Appl. Mater. Interfaces 2018, 10, 39228. 30226373 10.1021/acsami.8b12562

[243] A. Qu , M. Sun , J. Y. Kim , L. Xu , C. Hao , W. Ma , X. Wu , X. Liu , H. Kuang , N. A. Kotov , C. Xu , Nat. Biomed. Eng. 2021, 5, 103. 33106615 10.1038/s41551-020-00634-4

[244] M. Rahimzadegan , Q. Mohammadi , M. Shafieian , O. Sabzevari , Z. Hassannejad , Biomaterials Advances 2022, 134, 112634. 35577691 10.1016/j.msec.2021.112634

[245] K. I. Itzau‐Reid , et al., Adv. Funct. Mater. 2020, 30. 10.1002/adfm.202003710 PMC7610826 34035794

[246] H. Shao , T. Li , R. Zhu , X. Xu , J. Yu , S. Chen , L. Song , S. Ramakrishna , Z. Lei , Y. Ruan , L. He , Biomaterials 2018, 175, 93. 29804001 10.1016/j.biomaterials.2018.05.028

[247] N. Srivastava , V. Venugopalan , M. S. Divya , V. A. Rasheed , J. James , K. S. Narayan , Tissue Eng., Part A 2013, 19, 1984. 23544950 10.1089/ten.tea.2012.0626 PMC3725875

[248] M. H. Tan , X. H. Xu , T. J. Yuan , X. Hou , J. Wang , Z. H. Jiang , L. H. Peng , Biomaterials 2022, 283, 121413. 35276616 10.1016/j.biomaterials.2022.121413

[249] L. L. Wang , et al., ACS Biomater. Sci. Eng. 2019, 5, 613. 33405825 10.1021/acsbiomaterials.8b01481

[250] Y. Wu , Y. Peng , H. Bohra , J. Zou , V. D. Ranjan , Y. Zhang , Q. Zhang , M. Wang , ACS Appl. Mater. Interfaces 2019, 11, 4833. 30624894 10.1021/acsami.8b19631

[251] Q. Q. Xu , C. Li , S. Kuddannayai , Y. Zhang , RSC Adv. 2018, 8, 11027. 35541524 10.1039/c8ra01323c PMC9079102

[252] X. Z. Xu , et al., Frontiers in Bioengineering and Biotechnology 2022, 10. 10.3389/fbioe.2022.885746 PMC9213801 35757795

[253] J. Zhou , L. Cheng , X. Sun , X. Wang , S. Jin , J. Li , Q. Wu , Frontiers of Materials Science 2016, 10, 260.

[254] L. Zhou , L. Fan , X. Yi , Z. Zhou , C. Liu , R. Fu , C. Dai , Z. Wang , X. Chen , P. Yu , D. Chen , G. Tan , Q. Wang , C. Ning , ACS Nano 2018, 12, 10957. 30285411 10.1021/acsnano.8b04609

[255] W. Zhu , T. Ye , S. J. Lee , H. Cui , S. Miao , X. Zhou , D. Shuai , L. G. Zhang , Nanomedicine‐Nanotechnology Biology and Medicine 2018, 14, 2485. 28552650 10.1016/j.nano.2017.03.018

[256] M. Aliabouzar , L. G. Zhang , K. Sarkar , Sci. Rep. 2016, 6, 37728. 27883051 10.1038/srep37728 PMC5121887

[257] F. Assanah , H. Anderson , K. Grassie , L. Nair , Y. Khan , Regenerative Engineering and Translational Medicine 2024, 10, 205.

[258] Y. C. Cheng , C. W. Tsao , M. Z. Chiang , C. A. Chung , C. C. Chien , W. W. Hu , R. C. Ruann , C. Li , Microfluid. Nanofluid. 2015, 18, 587.

[259] V. Du , et al., Nat. Commun. 2017, 8. 10.1038/s41467-017-00543-2 PMC5596024 28900152

[260] Y. Gau , T. Li , Q. Sun , C. Ye , M. Guo , Z. Chen , J. Chen , B. Huo , Biomech. Model. Mechanobiol 2019, 18, 1731. 31115727 10.1007/s10237-019-01171-z

[261] S. M. Goldman , G. A. Barabino , BMC Biotechnol. 2016, 16. 26830345 10.1186/s12896-016-0240-6 PMC4736240

[262] T. Guo , L. Yu , C. G. Lim , A. S. Goodley , X. Xiao , J. K. Placone , K. M. Ferlin , B N. B. Nguyen , A. H. Hsieh , J. P. Fisher , Ann. Biomed. Eng. 2016, 44, 2103. 26577256 10.1007/s10439-015-1510-5 PMC4870157

[263] Y. C. Hao , M. H. Zong , Z. L. Wang , N. Li , Bioresources and Bioprocessing 2021, 8, 80. 38650256 10.1186/s40643-021-00435-w PMC10992857

[264] T. Kosawada , K. Ohnishi , H. Satoh , Z. G. Feng , K. Goto , Microsystem Technologies‐Micro‐and Nanosystems‐Information Storage and Processing Systems 2018, 24, 625.

[265] J. Kreutzer , M. Viehrig , R. P. Polonen , F. Zhao , M. Ojala , K. A. Setala , P. Kallio , Biomech. Model. Mechanobiol 2020, 19, 291. 31444593 10.1007/s10237-019-01211-8 PMC7005075

[266] L. Labusca , C. Danceanu , A. E. Minuti , D. D. Herea , A. Ghemes , C. Rotarescu , O. Dragos‐Pinzaru , M. Tibu , G. Marian , H. Chiriac , N. Lupu , Sci. Rep. 2022, 12, 16698. 36202902 10.1038/s41598-022-21145-z PMC9537172

[267] S. Lin , et al., Stem Cell Research & Therapy 2017, 8. 10.1186/s13287-017-0672-5 PMC5627486 28974254

[268] N. Nosoudi , C. Hart , I. McKnight , M. Esmaeilpour , T. Ghomian , A. Zadeh , R. Raines , J. E. R. Vick , Sci. Rep. 2021, 11, 24301. 34934143 10.1038/s41598-021-03824-5 PMC8692477

[269] A. Qu , M. Sun , J. Y. Kim , L. Xu , C. Hao , W. Ma , X. Wu , X. Liu , H. Kuang , N. A. Kotov , C. Xu , Nat. Biomed. Eng. 2021, 5, 103. 33106615 10.1038/s41551-020-00634-4

[270] S. D. Thorpe , C. T. Buckley , T. Vinardell , F. J. O'Brien , V. A. Campbell , D. J. Kelly , Ann. Biomed. Eng. 2010, 38, 2896. 20458627 10.1007/s10439-010-0059-6

[271] H. Yao , et al., J. Nanobiotechnol. 2018, 20.

[272] Z. Y. Yuan , et al., Sci. Rep. 2018, 8.29311689

[273] X. Hou , et al., Sci. Rep. 2016, 6.28442741

